# Ryanodine‐1‐Calstabin Complex Stabilizers in Antidoping Research: Synthesis, Metabolism, and Characterization

**DOI:** 10.1002/cplu.202500493

**Published:** 2025-12-27

**Authors:** Tristan Möller, Thomas Piper, Mario Thevis

**Affiliations:** ^1^ Institute of Biochemistry German Sport University Cologne Köln Germany; ^2^ European Monitoring Center for Emerging Doping Agents Cologne/Bonn Germany

**Keywords:** in vitro metabolism, LC‐HRMS, metabolite synthesis, RYR‐stabilizers

## Abstract

Awareness of new potential doping agents and the proactive implementation of detection methods are key aspects of preventive antidoping research. Ryanodine receptor‐1‐calstabin complex stabilizers (RYR‐stabilizers) are a novel class of drug candidates for the treatment of various diseases associated with leaky Ca^2+^ channels in the cardiac or skeletal muscle. Also, intense physical activity was shown to transiently cause leakage of skeletal muscle Ca^2+^ channels, and RYR‐stabilizers have been shown to restore normal activity and, thus, increase endurance performance. Consequently, such compounds are relevant targets in doping controls, and to date, in particular, compounds S107, JTV‐519, ARM 036, and ARM 210 have been subject of antidoping research. In this study, ARM 036 and ARM 210 as well as the commercially available compounds S107 and JTV‐519 were synthesized using a multistep approach. Subsequently, all compounds were investigated concerning their in vitro metabolic behavior, and various metabolites were identified. Selected metabolites were then chemically synthesized for comprehensive structure confirmation. The findings of this study will contribute to routine doping control analytical programs and allow for improving existing detection methods.

## Graphical abstract

RYR‐stabilizers such as S107, JTV‐519, ARM 036, and ARM 210 are emerging doping‐relevant compounds due to their performance‐enhancing effects on leaky skeletal muscle Ca^2+^ channels. To support proactive antidoping efforts, all four compounds were synthesized and their in vitro metabolism was systematically investigated. Multiple metabolites were identified, and selected structures were chemically synthesized for confirmation. These results expand analytical knowledge of RYR‐stabilizers and enable improved detection strategies for routine doping‐control programs.

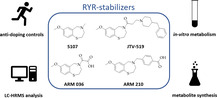

## 1 Introduction

With the continuous development of new drug candidates for, e.g., the treatment of orphan diseases or for improving pharmacological properties, new compounds that warrant consideration in the context of doping controls have constantly emerged. The World Anti‐Doping Agency (WADA) has featured the S0 category for unauthorized substances on its Prohibited List since 2011, which covers several of these novel pharmaceuticals [1].

Among these, the group of ryanodine receptor‐1‐calstabin complex stabilizers (RYR‐stabilizers) is mentioned, which is being investigated as a potential treatment option for Ryanodine receptor 1 (RYR 1)‐related myopathies, heart failure, cardiac arrhythmia, and Duchenne muscular dystrophy [2, 3, 4, 5]. These conditions are linked to leaky Ca^2+^ channel proteins, which occur either in the cardiovascular system (RYR 2) or in the skeletal muscle (RYR 1). Under physiological circumstances, these channel proteins remain either closed or open, releasing Ca^2+^ from the sarcoplasmic reticulum into the cytosol, which is crucial for inducing an action potential and muscle contraction [6, 7].

Under diseased conditions, but also during intense physical activity, RYR shows depletion of its calstabin subunit, resulting in a reduced formation of the action potential and leading to cardiac arrhythmia and muscle fatigue [8, 9]. As the name implies, RYR‐stabilizers stabilize such binding and, therefore, maintain the normal function of the Ca^2+^ channels even under diseased conditions or intense physical activity [10, 11, 12].

Representative compounds such as S107, JTV‐519, ARM 036, and ARM 210 have been discussed to exhibit such properties, warranting investigations into strategies how to determine their use in sports drug testing programs [4, 5, 9, 13]. While structural characteristics and analytical methods for S107 and JTV‐519 have already been reported, the target compounds ARM 036 and ARM 210 have not yet been studied with regard to adequate testing procedures and target metabolites [14, 15]. Besides aiming at analyzing the intact compounds, knowledge of their metabolic behavior is crucial for a comprehensive screening of doping control samples. And while data on the in vitro metabolism of S107 exist [16, 17], similar information on other representatives of the family of RYR‐stabilizers is currently not available.

Hence, the present study included the synthesis and mass spectrometric characterization of the compounds ARM 036 and ARM 210, followed by in vitro metabolism experiments using S107, JTV‐519, ARM 036, and ARM 210 to assess their biotransformation behavior. Observed metabolites were identified and investigated regarding their chemical structure by means of high resolution/high accuracy mass spectrometry and, in addition, selected metabolites were synthesized in order to validate their structures.

## 2 Results and Discussion

### 2.1 Synthesis of Parent Compounds

**SCHEME 1:**
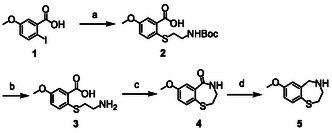
Synthesis of core structure **5**. Reaction conditions: (a) *tert*‐butyl (2‐mercaptoethyl)carbamate, Na_2_CO_3_, ethanedithiol, DMSO, 100°C, 12 h; (b) HCl in 1,4‐dioxane, RT, 72 h; (c) DCC, DMAP, DCM/DMF, 0°C to RT; 48 h; (d) LiAlH_4_, THF, reflux, 4 h [18, 19].

Since the herein investigated compounds share the common core structure of a 7‐methoxy‐benzothiazepine (compound **5**, see Scheme 1) while featuring different *N*‐linked substituents, **5** was first synthesized. Synthetic approaches similar to the one employed in this study have been described previously and were adapted as necessary [18, 19].

**SCHEME 2:**
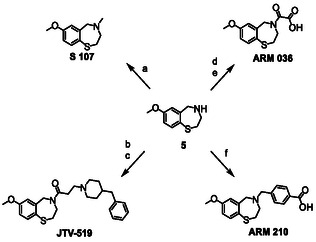
Synthesis of S107, JTV‐519, ARM 036, and ARM 210. Reaction conditions: (a) FA, formaldehyde, RT, 3 h; (b) 3‐bromopropanoyl chloride, TEA, DMF, 0°C to RT, 8 h; (c) 4‐benzylpiperidine, Na_2_CO_3_, DMF, 60°C, 3 h; (d) ethyl 2‐chloro‐2‐oxoacetate, pyridine, DCM, 0°C to RT, 8 h; (e) NaOH, MeOH/H_2_O, RT, 1.5 h; (f) 4‐(bromomethyl)benzoic acid, Na_2_CO_3_, DMF, RT, 8 h.

In brief, 2‐iodo‐5‐methoxybenzoic acid (**1**) was used as the starting material and was converted using *tert*‐butyl (2‐mercaptoethyl)carbamate and Na_2_CO_3_ to produce the boc‐protected amino derivative **2**. Compound **2** was then deprotected (**3**), using 4 M HCl in 1,4‐dioxane, followed by amidation to produce **4**. Reduction of compound **4** using LiAlH_4_ yielded the desired core structure **5**. The model compounds were then synthesized by treating **5** either with formaldehyde and formic acid (FA) to produce S107 or with 3‐bromopropanoyl chloride followed by treatment with 4‐benzylpiperidine to produce JTV‐519. ARM 036 and ARM 210 were prepared by reacting **5** either with ethyl 2‐chloro‐2‐oxoacetate followed by the deprotection of the ethyl group under alkaline conditions or with 4‐(bromomethyl)benzoic acid, respectively (Scheme 2).

All synthesized products were analyzed using both liquid chromatography high‐resolution mass spectrometry (LC‐HRMS) and nuclear magnetic resonance spectroscopy (NMR), suggesting the absence of by‐products of relevant abundance. A detailed mass spectrometric characterization of S107 and JTV‐519 can be found elsewhere [20]. The characterization of the new structures ARM 036 and ARM 210 is described below.

In MS^2^, ARM 036 (precursor ion at *m*/*z* 268.0639) exhibits the eliminations of either formic acid (46 u), CO_2_ (44 u) and CO (28 u) or, alternatively, the loss of glyoxylic acid (74 u), resulting in the formation of product ions at *m*/*z* 222.0586, 196.0793, and 194.0636, respectively. MS^3^ experiments further demonstrated the subsequent eliminations of either ammonia (17 u) or ethylene amine (43 u) from *m*/*z* 196.0793, yielding the most abundant product ion at *m*/*z* 153.0370 and the additional product ion at *m*/*z* 179.0527, respectively.

**SCHEME 3:**
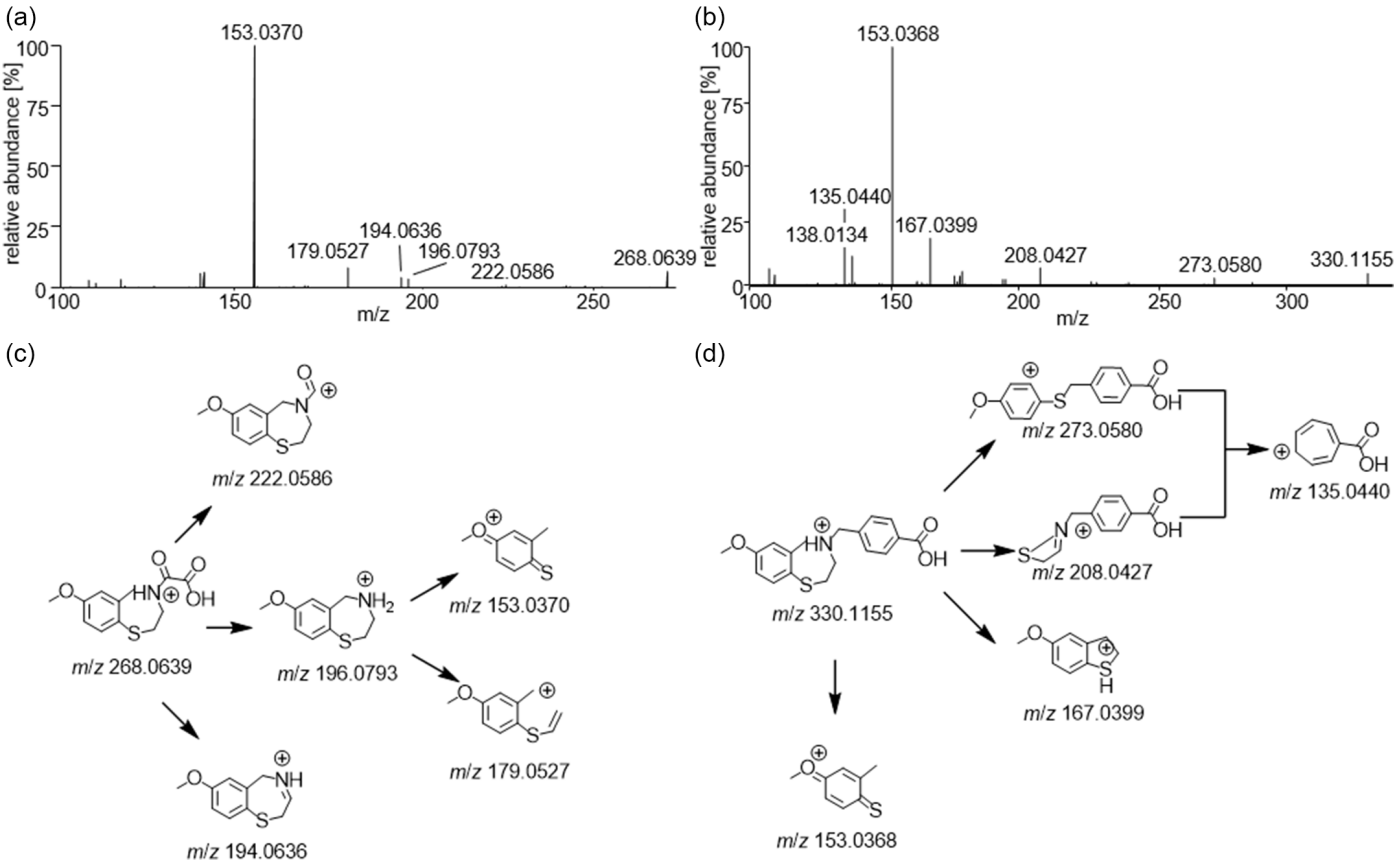
Mass spectrometric results for (a) ARM 036 and (b) ARM 210 also shown are the proposed dissociation pathway of the protonated molecules [M+H]^+^ of (c) ARM 036 and (d) ARM 210.

Similar to ARM 036, ARM 210 exhibits its most abundant product ion at *m*/*z* 153.0368. Additionally, a product ion at *m/z* 167.0399 is formed by the loss of C_9_H_11_NO_2_. Moreover, the cleavage of methoxy‐methylbenzene is suggested to result in the product ion at *m*/*z* 208.0427. ARM 210 also undergoes a rearrangement, producing a product at *m*/*z* 273.0580. MS^3^ experiments indicate that the ions at both *m*/*z* 208.0427 and *m*/*z* 273.0581 undergo further dissociation to form the common product ion at *m*/*z* 135.0440, which is tentatively assigned to the 4‐carboxycyclohepta‐2,4,6‐trien‐1‐ylium cation. The mass spectrometric results for ARM 036 and ARM 210 are shown in Scheme 3a,b, and an overview of the proposed dissociation pathways of both ARM 036 and ARM 210 is given in Scheme 3c,d. Structures of both substances were further confirmed using ^1^H, ^13^C APT and HSQC‐NMR.

### 2.2 In Vitro Metabolism Studies

All RYR‐stabilizers were investigated to determine their potential in vitro metabolic pathways. For this purpose, all compounds were incubated with human liver microsomes (HLMs). Also, to analyze phase‐II biotransformation reactions, respective cofactors were added to study the formation of glucuronic acid conjugates.

In the following sections, only mass spectrometric characterizations of those metabolites, for which confirmation via chemical synthesis is available, are presented. Further details on additional metabolites can be found in the supplementary data. Due to different outcomes in metabolic patterns, each RYR‐stabilizer will be discussed separately.

### 2.3 S107

**SCHEME 4:**
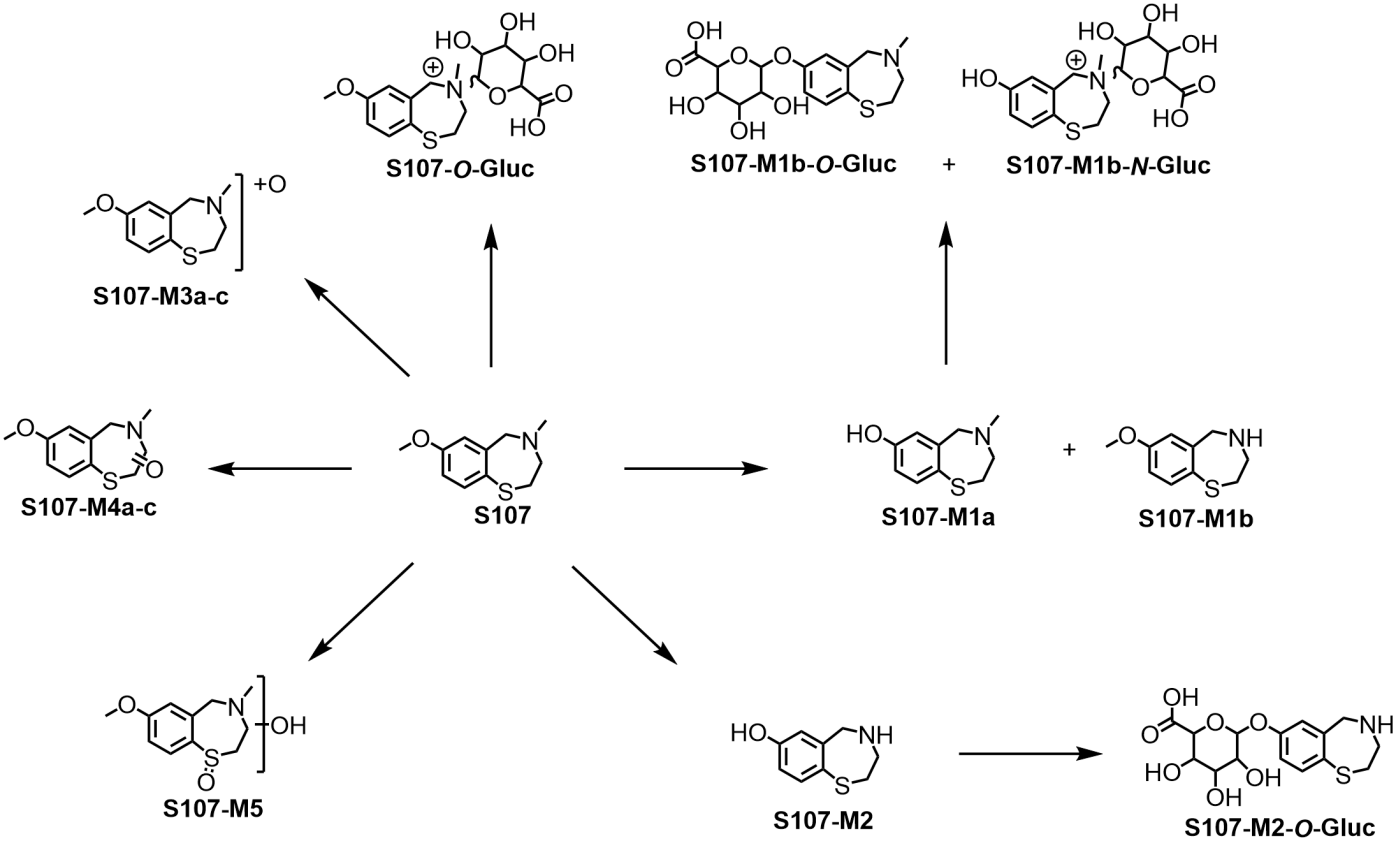
Overview of the in vitro metabolic pathways of S107.

The in vitro metabolic behavior of **S107** was previously investigated, and the results of the present study corroborate those reported in the literature [16]. **S107** yields demethylated metabolites (**S107‐M1a‐b**), with demethylations occurring either at the methoxy group of the benzothiazepine core structure (**S107‐M1a**) or at the amine (**S107‐M1b**) (Scheme 4).

Furthermore, **S107‐M1a** was shown to undergo glucuronidation, forming either *O*‐ or *N*‐glucuronides (**S107‐M1a‐*O*‐Gluc** and **S107‐M1a‐N‐Gluc**) [16]. (Detailed information for each metabolite can be found in the supplementary data).

**SCHEME 5:**
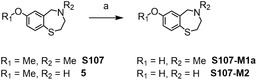
Synthesis of the potential metabolites S107‐M1a and S107‐M2. Reaction conditions: (a) BBr_3_, DCM, RT, 8 h.

In the previous work, metabolite **S107‐M1b** was synthesized and characterized [16]. Within this study, the synthesis of the *O‐*demethylated metabolite **S107‐M1a** using BBr_3_ is described as shown in Scheme 5. The presumed structure of the synthesized material was confirmed using ^1^H, ^13^C APT, and HSQC‐NMR. Both synthesized **S107‐M1a** and the in vitro generated metabolite displayed identical chromatographic and mass spectrometric behavior, corroborating the assigned composition of the analyte.

**SCHEME 6:**
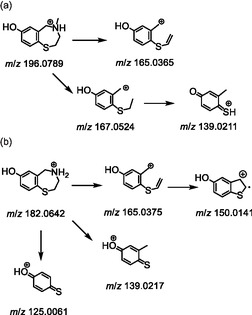
Proposed dissociation pathway of of the protonated molecules [M+H]^+^ of (a) **S107‐M1a** and (b) **S107‐M2**.

The presumed dissociation pathway has already been described in a previous study and is not discussed in further detail here. However, for the sake of completeness, an overview is illustrated in Scheme 6a [16].

Besides the metabolites described by Beuck et al., a total of six previously unreported metabolites were identified in this study. In addition to mono‐demethylated species, the di‐demethylated parent compound (*m*/*z* 182.0634) was also observed. As previously described, demethylation can be accomplished using BBr_3_. Thus, the putative metabolite **S107‐M2** was synthesized, starting from intermediate **5** (Scheme 5). Both, the synthesized material and the metabolite obtained in vitro exhibit similar chromatographic and mass spectrometric behavior. Further, the predicted structure was confirmed by means of ^1^H, ^13^C APT, and HSQC‐NMR.

The mass spectrometric characterization of the synthesized reference material in positive ionization mode (Scheme 6b) revealed a prominent peak at *m*/*z* 139.0217, which is suggested to represent the (2‐methyl‐4‐oxocyclohexa‐2,5‐dien‐1‐ylidene)sulfonium cation. This ion was identified as a characteristic product ion for the group of *O*‐demethylated RYR‐stabilizer metabolites. Furthermore, the precursor ion exhibited the loss of ammonia (17 u), yielding a product ion at *m*/*z* 165.0375, which subsequently eliminates a methyl radical (15 u) to produce an ion at *m*/*z* 150.0141. Additionally, the loss of a methyl ethylene amine‐radical (56 u) is postulated from the available data, forming the product ion at *m*/*z* 125.0061.

In the phase‐II metabolism experiments of S107, the corresponding glucuronide metabolite (**S107‐M2‐*O*‐Gluc**) was additionally identified.

**FIGURE 1:**
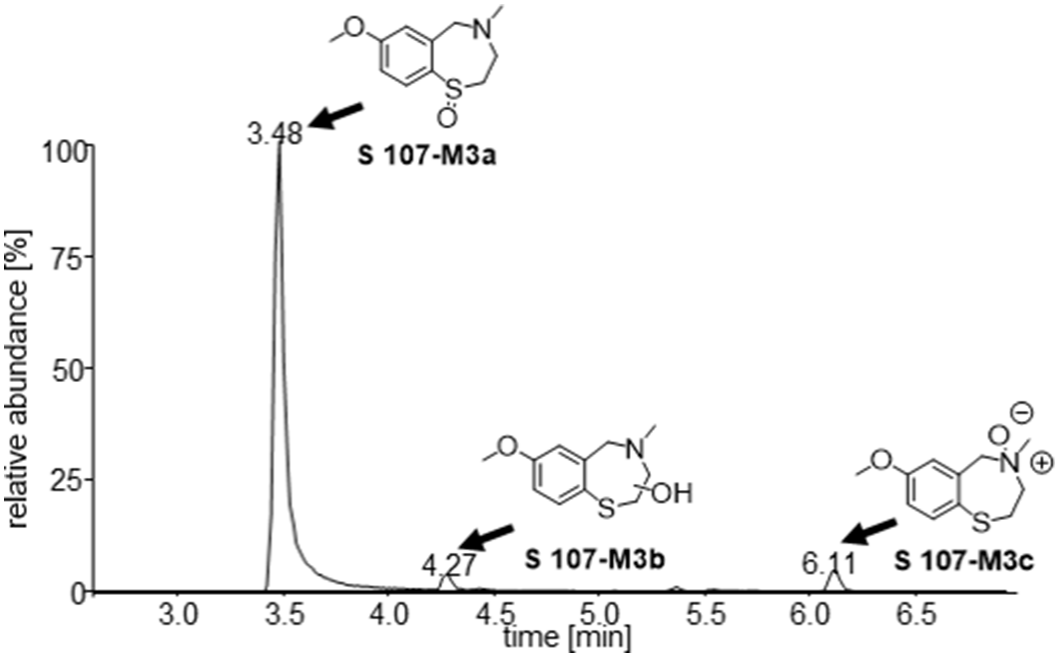
Extracted ion chromatogram of *m/z* 226.0896, representing the protonated molecules ([M+H]^+^) of the *N*‐, *S*‐oxidized or hydroxylated metabolites **S107‐M3a‐c**.

The metabolites referred to as **S107‐M3a** and **S107‐M3c**, exhibiting oxidation either at the nitrogen (Figure 1, *t* = 6.11  min) or sulfur moiety (Figure 1, *t* = 3.48 min) of the molecule has already been described in the literature ([M+H]^+^ = 226.0896 *m*/*z*) [16]. Interestingly, an additional signal with the same high resolution mass‐to‐charge ratio (Figure 1, *t* = 4.27 min) was detected. Based on this observation, occurrence of an additional hydroxylation of the S107 (**S107‐M3b**) was proposed.

**FIGURE 2:**
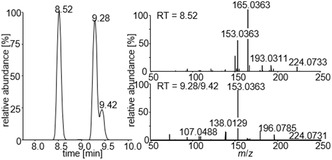
Chromatographic and mass spectrometric results for the tentatively identified *C*‐oxidized metabolites **S107‐M4a‐c** (*m*/*z *= 224.0740).

Furthermore, oxidized products with a mass shift of 2 u compared to the *N*‐, *S*‐oxidized or hydroxylated metabolites (*m*/*z *= 224.0740) were identified. These findings may be attributed to the formation of *C*‐oxidized metabolites. A total of three metabolites were obtained for this transformation, two of which exhibited similar mass spectra (*t* = 9.28 min and *t* = 9.42 min). This observation suggests the formation of closely related isomers (Figure 2).

An overview of the metabolites detected for **S107** is provided in Scheme 4.

### 2.4 JTV‐519

JTV‐519 was found to be extensively metabolized under the conditions applied in this work. The resulting biotransformations led to a wide range of metabolites, which are partly described in the following.

**SCHEME 7:**
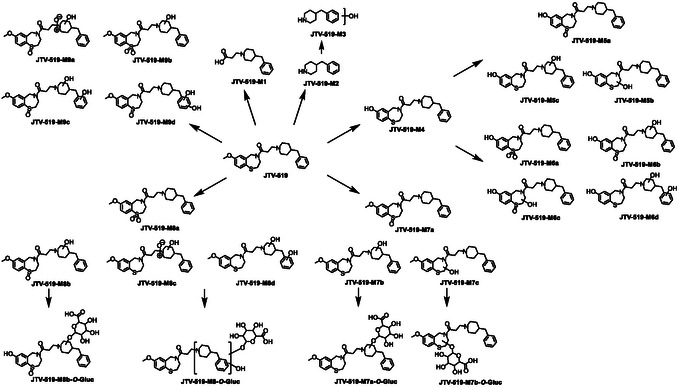
Overview of the in vitro metabolic pathway of **JTV‐519**.

The molecule undergoes cleavage of the *N‐*linked side chain through either amide cleavage, producing metabolite **JTV‐519‐M1** (*m*/*z* 248.1651) or through cleavage of the amine moiety, generating metabolite **JTV‐519‐M2** (*m*/*z* 176.1438). In the case of amine cleavage, also a hydroxylated analog, designated as **JTV‐519‐M3** (*m*/*z* 192.1390), was identified. All tentatively determined metabolites are summarized in Scheme 7.

**SCHEME 8:**
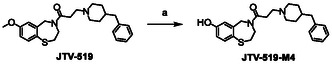
Synthesis of the potential metabolite JTV‐519‐M4. Reaction conditions: (a) boron tribromide, DCM, RT, 8 h.

Similar to S107, JTV‐519 undergoes *O*‐demethylation, forming metabolite **JTV‐519‐M4** (*m*/*z* 411.2108). Again, this metabolite was synthesized by demethylating its corresponding parent compound using BBr_3_ (Scheme 8). The structure of the synthesized reference material was confirmed using ^1^H, ^13^C APT, and HSQC‐NMR, and the chromatographic and mass spectrometric data of the reference material were compared with those of the in vitro generated metabolite to validate its structure. The MS^2^ spectrum obtained for the reference material revealed the formation a single product ion at *m*/*z* 188.1434, which is attributable to 4‐benzyl‐1‐methylenepiperidin‐1‐ium (C_13_H_18_N^+^). This product ion appears to result from the cleavage of the protonated molecule's side chain, a dissociation pathway that was found to be characteristic for JTV‐519 and its metabolites. As shown below, this product ion—or structurally related analogs—can be found in various metabolites.

JTV‐519 and its metabolite **JTV‐519‐M4** undergo extensive oxidation, resulting in a variety of metabolites. To determine the nature of these metabolites, their MS^2^ spectra were analyzed, and acetylation experiments were conducted to gain further insights into their chemical structures.

As mentioned above, the cleavage of the side chain, yielding the product ion at *m*/*z* 188.1434 or its corresponding mono‐oxidized (*m*/*z* 204.1390) and bis‐oxidized (*m*/*z* 220.1332) analogs, represents a characteristic dissociation pathway. These product ions were used to flag oxidative modifications within the molecule, and subsequent acetylation experiments were performed to further define the nature of these metabolic reactions. While hydroxyl groups are transformed into their corresponding acetates, *N*‐ and *S*‐oxides remain unchanged. Additionally, acetylated metabolites may exhibit improved chromatographic separation, whereas their unacetylated counterparts may not be fully separated using LC or show coelution with other metabolites. Due to the complexity and high number of the resulting metabolites, only selected metabolites are described here to illustrate the analytical workflow.

**FIGURE 3:**
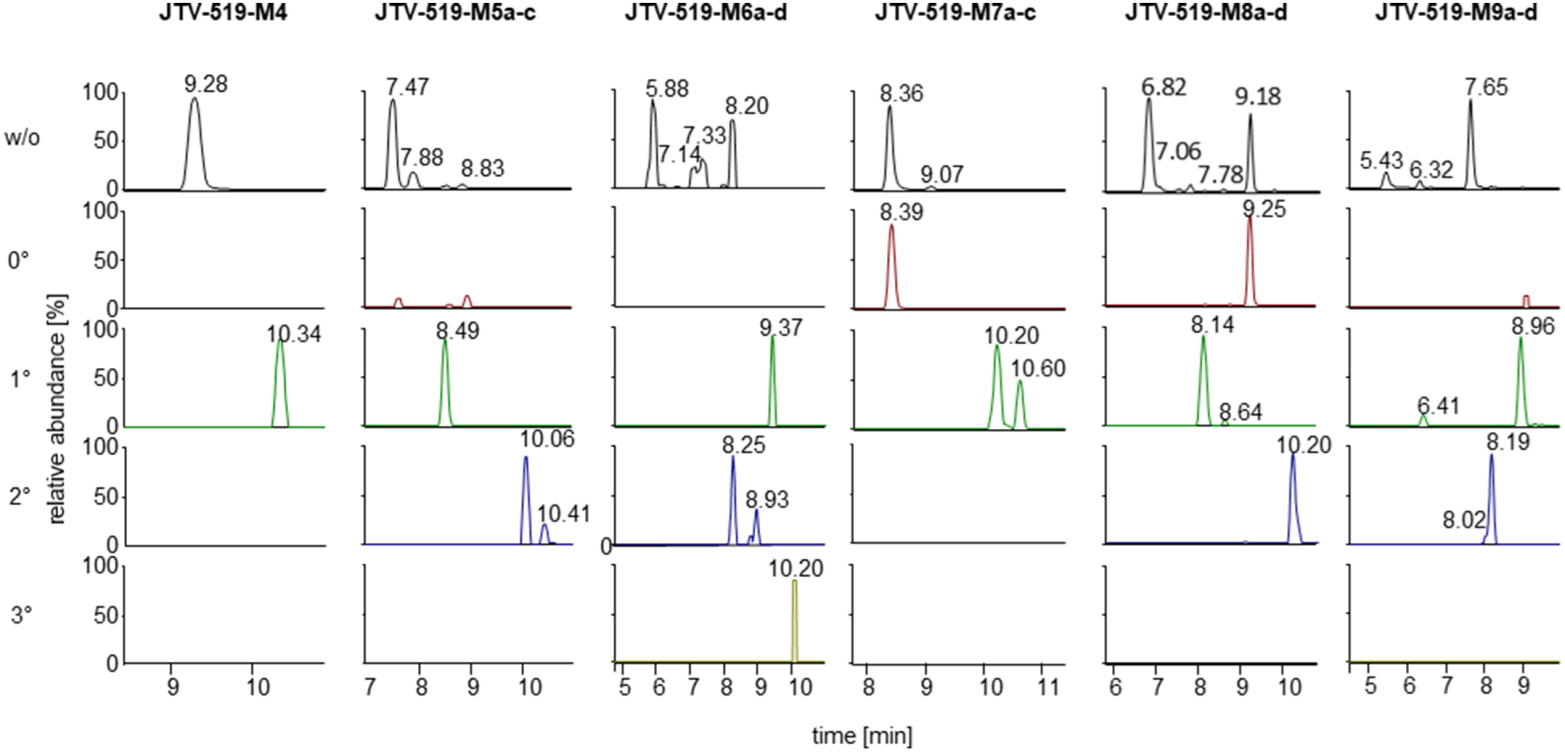
Acetylation pattern of the oxidized metabolites of JTV‐519. Shown are the extracted ion chromatograms of the suspected metabolites and their acetylated analogs. First row: extracted ion chromatograms of the nonacetylated metabolites; second row: extracted ion chromatograms of nonacetylated metabolites existing after acetylation; third row: extracted ion chromatograms of the mono‐acetylated metabolites; fourth row: extracted ion chromatograms of the bis‐acetylated metabolites; fifth row: extracted ion chromatograms of the tris‐acetylated metabolites.

The structure of metabolite **JTV‐519‐M4** was confirmed via chemical synthesis and is known to contain one hydroxyl group within the molecule. Therefore, it served as an internal quality control for the acetylation experiment. As shown in Figure 3, after acetylation, no unacetylated metabolite remains in the mixture, indicating a successful derivatization.

The group of metabolites subsumed under **JTV‐519‐M6** (*m*/*z *= 443.2014) refers to the demethylated and bis‐oxidized metabolites, which are used as example to illustrate the information generated by means of the acetylation experiment. In the native state (preacetylation, see Figure 3), four chromatographic signals were found, for two of which (at *t* = 7.14 min and *t* = 7.33 min) only modest separation is accomplished. In the MS^2^ spectrum generated from the precursor ion [M+H]^+^ of the first signal at *t *= 5.88 min, a product ion is observed at *m*/*z* 204.1390, indicating one oxidation occurring on the benzylpiperidine ring, while the second oxidation is plausibly located on the core structure of the molecule. Conversely, the MS^2^ spectra generated from the analytes corresponding to the peaks at *t* = 7.14 and *t *= 8.20 min exhibited a predominant peak at *m*/*z* 188.1434, suggesting both oxidations to exist on the core structure of the molecule. The peak at *t *= 7.33 min formed a product ion at *m*/*z *= 220.1339, indicating that both oxidative modifications occur on the benzylpiperidine ring.

After acetylation, four distinct chromatographic signals were observed, each corresponding to a different acetylation pattern, and the quality of separation was notably improved. Besides the expected acetylation of the demethylated methoxy group, the first signal at *t *= 9.37 min (**JTV‐519‐M6a**) showed no additional acetylation, indicating the absence of any metabolically introduced hydroxylation. The MS^2^ spectrum revealed a major product ion at *m*/*z* 188.1437, confirming that both oxidations are located on the core structure and suggesting the formation of a sulfone functional group. The analytes giving rise to the second and third signals, at *t *= 8.25 min (**JTV‐519‐M6b**) and *t *= 8.93 min (**JTV‐519‐M6c**), respectively, contain one additional acetate residue, indicating the presence of both hydroxylation and oxidation within the metabolites. The MS^2^ spectra suggest the oxidation of the pharmacophore, leading to the formation of a sulfoxide function in both metabolites. For **JTV‐519‐M6b**, MS^2^ data indicate a hydroxylation of the benzylpiperidine ring, most likely on the piperidine moiety, whereas **JTV‐519‐M6c** appears to be hydroxylated on the core structure. The fourth signal at *t *= 10.02 min produced a major product ion at *m*/*z *= 304.1551, attributable to the bis‐acetylated benzylpiperidine, suggesting that both oxidation reactions correspond to the introduction of hydroxyl groups.

The remaining metabolites were analyzed similarly, covering a wide range of biotransformation reactions such as hydroxylation, sulfoxidation, *N*‐oxidation, and sulfone formation.

A comprehensive overview of the chromatographic results with and without acetylation is shown in Figure 3, while Table 1 provides the mass spectrometric data after acetylation.

**TABLE 1:** Summary of mass spectral data of JTV‐519 metabolites obtained after acetylation. For the intact analyte, calculated values were used, while the values for the *m*/*z* after acetylation and the corresponding product ions show the experimental data with an acceptance of 5 ppm.

Species	Protonated molecule of intact analyte, *m*/*z*	Metabolite	Retention time, min	N° acetate groups	Mass‐to‐charge ratio after acetylation, *m*/*z*	Product ions, *m*/*z*	Deduced formula
Demethylation	411.2101	**JTV‐519‐M4**	10.34	1	453.2206	188.1439	C_13_H_18_N^+^
Demethylation + mono‐ oxidation	427.2059	**JTV‐519‐M5a**	8.50	1	469.2150	222.0587	C_11_H_12_NO_2_S^+^
						188.1436	C_13_H_18_N^+^
	427.2059	**JTV‐519‐M5b**	10.06	2	511.2256	246.1495	C_15_H_20_NO_2_^+^
						186.1282	C_13_H_16_N^+^
	427.2059	**JTV‐519‐M5c**	10.44	2	511.2256	188.1438	C_13_H_18_N^+^
Demethylation + bis‐ oxidation	443.2014	**JTV‐519‐M6a**	9.37	1	485.2105	188.1437	C_13_H_18_N^+^
	443.2014	**JTV‐519‐M6b**	8.25	2	527.2210	467.2010	C_26_H_31_N_2_O_4_S^+^
						246.1494	C_15_H_20_NO_2_^+^
						222.0589	C_11_H_12_NO_2_S^+^
	443.2014	**JTV‐519‐M6c**	8.93	2	527.2210	280.0645	C_13_H_14_NO_4_S^+^
						188.1437	C_13_H_18_N^+^
	443.2014	**JTV‐519‐M6d**	10.02	3	569.2332	304.1551	C_17_H_22_NO_4_^+^
Mono‐ oxidation	441.2206	**JTV‐519‐M7a**	8.39	0	441.2206	188.1437	C_13_H_18_N^+^
	441.2206	**JTV‐519‐M7b**	10.20	1	483.2312	246.1495	C_15_H_20_NO_2_^+^
						186.1282	C_13_H_16_N^+^
	441.2206	**JTV‐519‐M7c**	10.60	1	483.2312	188.1437	C_13_H_18_N^+^
Bis‐oxidation	457.2156	**JTV‐519‐M8a**	9.24	0	457.2156	188.1438	C_13_H_18_N^+^
	457.2156	**JTV‐519‐M8b**	8.14	1	499.2261	439.2060	C_25_H_31_N_2_O_3_S^+^
						246.1492	C_15_H_20_NO_2_^+^
						194.0637	C_10_H_12_NOS^+^
	457.2156	**JTV‐519‐M8c**	8.64	1	499.2261	439.2060	C_25_H_31_N_2_O_3_S^+^
						262.1446	C_15_H_20_NO_3_^+^
						202.1232	C_13_H_16_NO^+^
	457.2156	**JTV‐519‐M8d**	10.21	2	541.2367	304.1549	C_17_H_22_NO_4_^+^
						246.1496	C_15_H_20_NO_2_^+^
Tris‐oxidation	473.2105	**JTV‐519‐M9a**	6.41	1	515.2210	455.2012	C_25_H_31_N_2_O_4_S^+^
						262.1446	C_15_H_20_NO_3_^+^
						202.1232	C_13_H_16_NO^+^
	473.2105	**JTV‐519‐M9b**	8.96	1	515.2210	455.2006	C_25_H_31_N_2_O_4_S^+^
						246.1494	C_15_H_20_NO_2_^+^
						186.1282	C_13_H_16_N^+^
	473.2105	**JTV‐519‐M9c**	8.02	2	557.2316	497.2115	C_27_H_33_N_2_O_5_S^+^
						304.1556	C_17_H_22_NO_4_^+^
						244.1342	C_15_H_18_NO_2_^+^
	473.2105	**JTV‐519‐M9d**	8.20	2	557.2316	304.1553	C_17_H_22_NO_4_^+^
						194.0635	C_10_H_12_NOS^+^

This study also investigated the phase‐II metabolism, with a focus on the formation of glucuronides. Two primary oxidized metabolites, **JTV‐519‐M7** (mono‐oxidation) and **JTV‐519‐M8** (bis‐oxidation) were found to undergo subsequent glucuronidation. For **JTV‐519‐M7**, a total of four potential glucuronides was identified, of which two signals were concluded to result from hydroxylation and glucuronidation on the core structure. The remaining two signals are proposed to represent the hydroxylation and glucuronidation of the benzlypiperidine moiety. For **JTV‐519‐M8** (bis‐oxidation), four corresponding glucuronides were detected.

In total, this study identified 22 phase‐I metabolites and 8 phase‐II metabolites. A comprehensive overview of all identified metabolites is provided in Scheme 7.

### 2.5 ARM 036 and ARM 210

**SCHEME 9:**
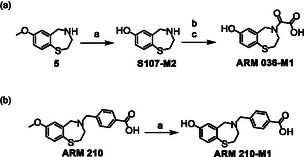
Synthesis of the potential metabolites (a) **ARM 036‐M1** and (b) **ARM 210‐M1**. Reaction conditions: (a) BBr_3_, DCM, RT, 8 h; (b) ethyl 2‐chloro‐2‐oxoacetate, pyridine, DCM, 0°C to RT, 8 h; (c) NaOH, MeOH/H_2_O, RT, 12 h.

Under the conditions applied in this study, only few metabolites were generated from ARM 036 and ARM 210. As before, *O‐*demethylation was observed and, for comparison, the resulting metabolites, **ARM 036‐M1** and **ARM 210‐M1**, were synthesized using BBr_3_ as demonstrated for S107 and JTV‐519 (Scheme 9). However, due to the instability of ARM 036 and its presumed metabolites, the core structure **5** was first demethylated (to produce the potential metabolite **S107‐M2**) before the oxalyl group was added (Scheme 9a). The structures of the synthesized material were confirmed using ^1^H, ^13^C APT, and HSQC‐NMR.

Both synthesized compounds show strong concordance in LC and MS with their in vitro generated counterparts. Therefore, the structure of the in vitro generated metabolites was considered confirmed and an in‐depth MS characterization was conducted.

In both cases, a product ion at *m*/*z* 139.0213 was identified as the predominant dissociation product, as previously described for analog metabolites such as **S107‐M1b** and **S107‐M2**. However, metabolite **ARM 036‐M1** also eliminated formic acid (46 u), forming a product ion at *m*/*z* 208.0436, and the loss of the amino‐oxoacetic acid group (89 u) was suggested to result in the product ion at *m*/*z* 165.0371.

**SCHEME 10:**
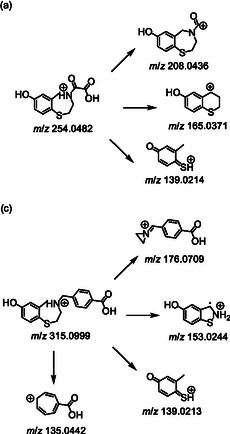
Proposed dissociation pathway of the protonated molecules [M+H]^+^ of (a) **ARM 036‐M1** and (b) **ARM 210‐M1**.

Similar to the parent compound, **ARM 210‐M1** gave rise to an intense signal at *m*/*z* 135.0442 that is suggested to represent the 4‐carboxycyclohepta‐2,4,6‐trien‐1‐ylium cation. Furthermore, the protonated molecule yielded a product ion at *m*/*z* 153.0244, which can be attributed to a benzothiazolidine cation. Additionally, it undergoes dissociation of a benzothioether function, forming a product ion at *m*/*z* 176.0709. An overview of the proposed dissociation pathways of both metabolites can be found in Scheme 10.

For both, **ARM 036** and **ARM 210** oxidized/hydroxylated species were obtained (*m*/*z* 346.1093 and *m*/*z* 284.0576, respectively). As already described for JTV‐519, acetylation experiments were conducted to corroborate the postulated chemical nature of these compounds. In both cases, no acetylation was observed, indicating the formation of oxidized metabolites such as sulfoxides. In the case of **ARM 210**, a corresponding glucuronide to the above‐described metabolite **ARM 210‐M1** was identified (**ARM 210‐M1‐*O*‐Gluc**; *m*/*z *= 492.1323).

**SCHEME 11:**
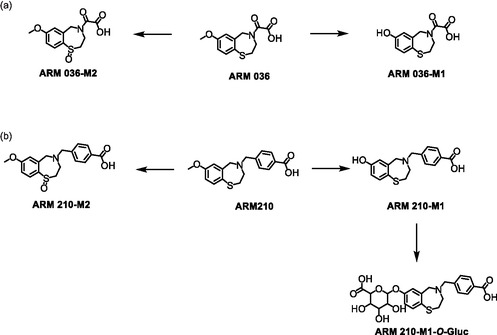
Overview of the in vitro metabolic pathways of (a) ARM 036 and (b) ARM 210.

An overview of the identified metabolites can be found in Scheme 11.

## 3 Conclusion

This work presents an in‐depth analysis of the in vitro metabolic pathways of selected RYR‐stabilizers. Despite their common core structure, the identified metabolic pathways differ between the compounds, not only in the number of metabolites formed but also in the specific biotransformations they undergo. However, oxidation and demethylation were observed for each compound. The demethylated metabolites of each RYR‐stabilizer were synthesized by eliminating the methyl group using BBr_3_, and the resulting products were used to confirm the postulated structures of the in vitro generated metabolites.

The results of this study represent the basis on which potential metabolites may be implemented into routine doping control analytical methods and will contribute to improving existing testing procedures. Further studies on the in vivo metabolism of RYR‐stabilizers will be necessary in the future to ensure the sensitive detection of their misuse.

## 4 Experimental Section

### 4.1 Reagents and Chemicals

2‐Iodo‐5‐methoxybenzoic acid and *tert*‐butyl (2‐mercaptoethyl)carbamate were purchased by BLDPharm (Reinbek, Germany). 4‐Benzylpiperidine, dimethylformamide (DMF), *N*,*N′*‐dicyclohexylcarbodiimide (DCC), ethane‐1,2‐dithiol, formaldehyde solution (37% in H_2_O), HCl solution (4 M in 1,4‐dioxane), lithium aluminum hydride solution (2 M in THF), magnesium chloride (MgCl_2_), potassium dihydrogen phosphate (KH_2_PO_4_), pyridine, sodium carbonate (Na_2_CO_3_), sodium hydroxide (NaOH), D‐saccharic acid‐1,4‐lactone (SL), and uridine diphosphate glucuronic acid (UDGPA) were obtained from Sigma–Aldrich (St. Louis, Missouri, USA). Dimethyl sulfoxide (DMSO), dichloromethane (DCM), and HLMs were obtained from Thermo Scientific (Bremen, Germany). Celite, 4‐dimethylaminopyridine (DMAP), magnesium sulfate (MgSO_4_), triethyl amine (Et_3_N), and tetrahydrofuran (THF) were purchased from Carl Roth (Karlsruhe, Germany). Acetic anhydride (Ac_2_O), ethyl chloro oxoacetate, 3‐bromopropanoyl chloride, and 4‐(bromomethyl)benzoic acid were obtained from Merck (Darmstadt, Germany). Acetonitrile (ACN), ethyl acetate (EtOAc), formic acid (FA), and *n*‐pentane were obtained from VWR Chemicals (Langenfeld, Germany). Boron tribromide (BBr_3_) was used from Alfa Aesar (Haverhill, Massachusetts, USA), and the nicotinamide adenine dinucleotide phosphate (NADPH) regenerating system was purchased form Promega (Madison, Wisconsin, USA). MeOH was purchased from J.T.Baker (Phillipsburg, New Jersey, USA). Hydrogen gas (99.999%) was from Praxair (Düsseldorf, Germany). Ultrapure water was received from a Barnstead GenPure xCAD Plus from Thermo Scientific (Bremen, Germany).

Column chromatography was performed using silica gel (63–200 µm) from Supelco (Sigma–Aldrich, St. Louis, Missouri, USA). For reaction control and control of the column chromatography, thin layer chromatography plates were used from Merck (Darmstadt, Germany). Chromabond C18 6cc SPE cartridges were purchased from Macherey–Nagel (Düren, Germany).

### 4.2 NMR Spectroscopy

NMR spectra were acquired using a Bruker Avance I 300 and Bruker Avance III 499. ^1^H NMR spectra were acquired at a frequency of 300.1 or 499.9 MHz, while ^13^C NMR spectra were acquired at a frequency of 125.7 MHz. Peak assignments were assisted with two‐dimensional spectra (H,H‐COSY, H,C‐HMBC, and H,C‐HMQC). The chemical shift *σ* and the coupling constant 3J or 4J are indicated in ppm and in Hz, respectively. The multiplicity is classified as singlet (s), doublet (d), triplet (t), doublet doublet (dd), triplet triplet (tt), and multiplet (m).

### 4.3 Synthesis

#### 4.3.1 2‐((2‐((*tert.*‐Butoxycarbonyl)amino)ethyl)thio)‐5‐methoxybenzoic acid (2)

In a baked‐out flask, 2‐iodo‐5‐methoxybenzoic acid (**1**) (3.00 g; 10.8 mmol; 1.0 eq) was dissolved in DMSO (20 mL; 2 mL/mmol) and *tert*‐butyl (2‐mercaptoethyl)carbamate (2.10 g; 11.9 mmol; 1.1 eq) and Na_2_CO_3_ (2.28 g; 21.6 mmol; 2.0 eq) were added. After the addition of ethane‐1,2‐dithiol (0.15 mL; 0.2 mmol; 0.16 eq), the reaction mixture was stirred over night at 100°C. The reaction was cooled to room temperature (RT) and was then filtrated over Celite. The solvent was removed under reduced pressure, and the crude product was purified using column chromatography (*n‐*pentane:EtOAc = 5:1). The final product was isolated as a beige solid with a yield of 2.96 g (83.7%).

^1^H NMR (DMSO‐d_6_; 500 MHz): *δ* 1.38 (s, 9H), 2.92 (t; *J *= 7.2 Hz, 2H), 3.12 (q; *J *= 6.7 Hz, 2H), 3.78 (s, 3H), 7.12 (dd; *J *= 2.8, 8.7 Hz, 1H), 7.35 (d; *J *= 2.9 Hz, 1H), 7.42 (d; *J *= 8.7 Hz, 1H).

^13^C NMR (DMSO‐d_6_; 125 MHz): *δ* 28.82 (CH_3_), 32.27 (CH_2_), 38.11 (*C*_quat_.), 40.18 (CH_2_), 55.93 (CH_3_), 78.24 (*C*_quat_.), 115.77 (CH), 118.84 (CH), 127.56 (*C*_quat._), 129.32 (CH), 157.04 (*C*_quat._), 159.63 (*C*_quat._), (168.34 (*C*_quat._).

#### 4.3.2 2‐((2‐Aminoethyl)thio)‐5‐methoxybenzoic acid (3)

**2** (2.96 g; 9.04 mmol; 1.0 eq) was dissolved in HCl in 1,4‐dioxane (4 M; 7.0 mL) and was stirred at RT for 72 h. The resulting suspension was filtrated and the solvent was removed in vacuo. The resulting crude product was isolated as a white solid (2.09 g) that was used without further purification.

^1^H NMR (DMSO‐d_6_; 500 MHz): *δ* 3.02 (t; *J *= 5.9 Hz, 2H), 3.14 (q; *J *= 5.9 Hz, 2H), 3.79 (s, 3H), 7.01 (dd; *J *= 2.9, 8.4 Hz, 1H), 7.05 (d; *J* = 2.9 Hz, 1H), 7.40 (d; *J* = 8.4 Hz, 1H).

^13^C NMR (DMSO‐d_6_; 125 MHz): δ 29.53 (CH_2_), 34.42 (*C*_quat._), 38.74 (CH_2_), 56.81 (CH_3_), 115.11 (CH), 118.08 (CH), 127.56 (*C*_quat._), 129.05 (CH), 157.54 (*C*_quat._), 168.34 (*C*_quat._).

#### 4.3.3 7‐Methoxy‐3,4‐dihydrobenzo[*f*][1,4]thiazepine‐5(2*H*)‐one (4)

For cyclization, **3** (2.09 g; 9.0 mmol; 1.0 eq) was added to a mixture of DCM (18 mL; 2 mL/mmol) and DMF (9 mL; 1 mL/mmol); afterward, DMAP (0.11 g; 0.9 mmol; 0.1 eq) and DCC (2.25 g; 10.8 mmol; 1.2 eq) were added at 0°C. The reaction mixture was warmed up to RT and was then stirred for 12 h. The reaction was stopped by the addition of H_2_O, and the aqueous phase was washed with EtOAc (3x). The combined organic phases were dried over MgSO_4_, and afterward, the solvent was removed under reduced pressure. The crude product was then purified using column chromatography (*n‐*pentane:EtOAc = 10:1), and afterward, the final product was isolated as a beige solid with a yield of 1.00 g (52.0%).

^1^H NMR (DMSO‐d_6_; 500 MHz): *δ* 3.01 (t; *J *= 5.9 Hz, 2H), 3.14 (q; *J *= 5.9 Hz, 2H), 3.79 (s, 3H), 7.01 (dd; *J *= 2.9, 8.4 Hz, 1H), 7.05 (d; *J *= 2.9 Hz, 1H), 7.40 (d; *J *= 8.4 Hz, 1H).

^13^C NMR (DMSO‐d_6_; 125 MHz): *δ* 36.25 (*C*_quat._), 37.17 (CH_2_), 39.62 (CH_2_), 55.93 (CH_3_), 114.93 (CH), 117.18 (CH), 119.74 (*C*_quat._), 135.62 (CH), 160.23 (*C*_quat._), 170.76 (*C*_quat._).

#### 4.3.4 7‐Methoxy‐2,3,4,5‐tetrahydrobenzo[*f*][1,4]thiazepine (5)

**4** (0.60 g; 2.9 mmol; 1.0 eq) was dissolved in THF (1.5 mL; 1.5 mL/mmol). The solution was cooled down to 0°C, and LiALH_4_ solution (1 M in THF; 5.73 mL; 7.7 mmol; 3.0 eq) was added. Afterward, the mixture was stirred under reflux for 3 h. For quenching, the reaction was cooled to 0°C and first MeOH and then H_2_O was added. The mixture was extracted with EtOAc (5x) and the combined organic layers were dried over MgSO_4_. After removing of the solvent under reduced pressure, the crude product was isolated as a beige solid with a yield of 0.48 g and was used without further purification.

^1^H NMR (DMSO‐d_6_; 500 MHz): *δ* 2.62 (t; *J *= 4.6 Hz, 2H), 3.18 (t; *J *= 4.1 Hz, 2H), 3.73 (s, 3H), 3.91 (s, 2H), 6.71 (dd; *J *= 2.9, 8.4 Hz, 1H), 6.88 (d; *J *= 2.7 Hz, 1H), 7.39 (d; *J *= 8.4 Hz, 1H).

^13^C NMR (DMSO‐d_6_; 125 MHz): *δ* 36.42 (CH_2_), 53.67 (CH_2_), 55.39 (CH_2_), 55.65 (CH_3_), 112.20 (CH), 115.77 (CH), 127.45 (*C*_quat._), 133.78 (CH), 149.63 (*C*_quat._), 159.22 (*C*_quat._).

#### 4.3.5 7‐Methoxy‐4‐methyl‐2,3,4,5‐tetrahydrobenzo[*f*][1,4]thiazepine (S107)

Core structure **5** (30 mg; 0.2 mmol; 1.0 eq) was mixed with FA (29 µL; 0.8 mmol; 4.0 eq) and formaldehyde (26 µL; 0.5 mmol; 2.5 eq), and the reaction mixture was stirred for 3 h at RT. The reaction was stopped by the addition of H_2_O and was then extracted with EtOAc (3x). After drying over MgSO_4_, the solvent was removed under reduced pressure. For purification, a Chromabond C18 SPE cartridge (6cc) was used. The SPE was conditioned with MeOH (3 mL) and H_2_O (3 mL). The crude product was dissolved in H_2_O (3 mL) and was then eluted with an ACN/H_2_O gradient (10% to 60% ACN). The eluent was evaporated using a N_2_ stream. The final product was isolated as a white solid with a yield of 12 mg (38.2%).

^1^H NMR (DMSO‐d_6_; 500 MHz): *δ* 2.48 (s, 3H), 2.85 (t; *J *= 4.3 Hz, 2H), 3.38 (t; *J *= 4.7 Hz, 2H), 3.77 (s, 3H), 4.25 (s, 2H), 6.86 (dd; *J *= 2.8, 8.4 Hz, 1H), 7.12 (d; *J *= 2.5 Hz, 1H), 7.44 (d; *J *= 8.4 Hz, 1H).

^13^C NMR (DMSO‐d_6_; 125 MHz): *δ* 30.19 (CH_2_), 52.41 (CH_3_), 55.83 (CH_3_), 59.57 (CH_2_), 60.47 (CH_2_), 114.04 (CH), 118.751 (CH), 128.01 (*C*_quat._), 133.96 (CH), 152.42 (*C*_quat._), 159.44 (*C*_quat._).

#### 4.3.6 3‐(4‐Benzylpiperidin‐1‐yl)‐1‐(7‐methoxy‐2,3‐dihydrobenzo[*f*][1,4]thiazepin‐4(5*H*)‐yl)propan‐1‐one (JTV‐519)

**5** (100 mg; 0.5 mmol; 1.0 eq) was dissolved in THF (5.0 mL; 10 mL/mmol), and then, 3‐bromopropanoyl chloride (103 µL; 1.0 mmol 2.0 eq) and Et_3_N (140 µL; 1.0 mmol; 1.0 eq) were added. The reaction mixture was stirred for 3 h at RT and was then stopped by the addition of H_2_O. The aqueous phase was extracted with EtOAc (3x), which was then dried over MgSO_4_. After evaporation of the solvent under reduced pressure, the crude product was dissolved in DMF (1 mL; 2 mL/mmol) and 4‐benzylpiperidine (133 µL, 0.8 mmol; 1.5 eq) and Na_2_CO_3_ (108 mg; 1.0 mmol; 2.0 eq) were added. The solution was stirred for 2 h at RT and was then diluted in H_2_O. The reaction mixture was extracted using EtOAc (3x) and was then dried over MgSO_4_. The solvent was evaporated in vacuo, and afterward, the crude product was purified using a Chromabond C18 SPE cartridge (6cc) with a gradient of ACN/H_2_O (10% to 75% ACN). The solvent was removed with a N_2_ stream and the final product was isolated as a colorless oil with a yield of 30 mg (13.9%).

^1^H NMR (DMSO‐d_6_; 500 MHz): *δ* 1.13 (m, 3H), 1.52 (m, 5H) 2.45 (m, 6H), 2.76 (m, 2H), 2.91 (m, 1H), 3.92 (s, 2H), 3.74 (m, 3H), 3.92 (m, 2H), 4.53 (s, 1H), 4.70 (s, 1H), 1H 6.77 (dd; *J *= 2.7, 8.4 Hz, 1H), 6.98 (d; *J *= 2.8 Hz, 1H), 7.16 (m, 6H).

^13^C NMR (DMSO‐d_6_; 125 MHz): *δ* 31.89 (CH_2_), 33.51 (CH_2_), 35.03 (CH_2_), 36.29 (CH_2_), 37.86 (CH), 41.43 (CH_2_), 42.54 (CH_2_), 45.61 (CH_2_), 55.73 (CH_3_), 58.013 (CH_2_), 112.82 (CH), 113.25 (CH), 116.91(CH), 117.98 (CH), 126.29 (CH), 126.64 (*C*_quat._), 129.55 (CH), 140.59 (*C*_quat._), 144.39 (*C*_quat._), 159.05 (*C*_quat._), 169.56 (*C*_quat._).

#### 4.3.7 2‐(7‐Methoxy‐2,3‐dihydrobenzo[*f*][1,4]thiazepin‐4(5*H*)‐yl)‐2‐oxoacetic acid (ARM 036)

To a solution of **5** (100 mg; 1.0 mmol; 1.0 eq) in DCM (10 mL; 10 mL/mmol) ethyl chloro oxoacetate (172 µL; 1.5 mmol; 1.5 eq) and pyridine (165 µL; 2.1 mmol; 2.0 eq) were added and the mixture was stirred for 1 h at RT. Then, the reaction was stopped by the addition of water and both phases were separated. The aqueous phase was extracted with EtOAc (3x), and the combined organic phases were dried over MgSO_4_. After removing of the solvent under reduced pressure, the intermediate product was dissolved in MeOH (3 mL; 10 mL/mmol) and NaOH (13 mg; 0.3 mmol; 1.0 eq) in H_2_O (3 mL; 10 mL/mmol) was added slowly. After 1.5 h, the reaction was stopped and extracted with EtOAc (3x). The combined organic phases were dried using MgSO_4_, and afterward, the solvent was removed in vacuo. The crude product was then purified using a Chromabond C18 SPE cartridge (6cc) that was eluted with ACN/H_2_O (10% to 75% ACN). After drying using a N_2_ stream, the final product was isolated as a white solid with a yield of 57 mg (64.2%).

^1^H NMR (DMSO‐d_6_; 500 MHz): *δ* 2.76 (t; *J *= 4.6 Hz, 2H), 3.75 (s, 3H), 3.79 (t; *J *= 3.6 Hz, 2H), 4.47 (s, 2H), 6.77 (dd; *J *= 2.9, 8.4 Hz, 1H), 6.98 (d; *J *= 2.7 Hz, 1H), 7.42 (d; *J *= 8.4 Hz, 1H).

^13^C NMR (DMSO‐d_6_; 125 MHz): *δ* 35.48 (CH_2_), 50.11 (CH_2_), 53.14 (CH_2_), 55.74 (CH_3_), 112.74 (CH), 117.77 (CH), 126.92 (*C*_quat._), 134.06 (CH), 144.83 (*C*_quat._), 159.10 (*C*_quat._), 167.60 (*C*_quat._), 170.25 (*C*_quat._).

#### 4.3.8 4‐((7‐Methoxy‐2,3‐dihydrobenzo[*f*][1,4]thiazepin‐4(5*H*)‐yl)methyl)benzoic acid (ARM 210)

To a solution of **5** (50 mg; 0.3 mmol; 1.0 eq) in DMF (300 µL; 1 mL/mmol) was added 4‐(bromomethyl)benzoic acid (55 mg; 0.3 mmol; 1.0 eq), and the resulting mixture was stirred for 4 h. After completion of the reaction (screened by LC‐HRMS), the reaction was stopped and then extracted with EtOAc (3x) followed by drying over MgSO_4_. Then, the solvent was removed under reduced pressure, and the crude product was purified with a Chromabond C18 SPE cartridge (6cc) that was eluted with ACN/H_2_O (10% to 50% ACN). The solvent was removed under a N_2_ stream, and the final product was then isolated as a colorless oil with a yield of 37 mg (43.0%).

^1^H NMR (DMSO‐d_6_; 500 MHz): *δ* 2.72 (m, 2H), 3.20 (m, 2H), 3.58 (s, 2H), 3.70 (s, 3H), 4.03 (s, 2H), 6.65 (d; *J *= 2.8 Hz, 1H), 6.77 (dd; *J *= 2.8, 8.4 Hz, 1H), 7.40 (d; *J *= 8.2 Hz, 1H), 7.43 (d; *J *= 8.1 Hz, 1H), 7.91 (d; *J *= 8.2 Hz, 1H).

^13^C NMR (DMSO‐d_6_; 125 MHz): *δ* 30.18 (CH_2_), 55.56 (CH_3_), 55.56 (CH_2_), 57.93 (CH_2_), 59.49 (CH_2_), 112.88 (CH), 117.18 (CH), 127.68 (*C*_quat._), 129.06 (CH), 129.78 (CH), 129.97 (*C*_quat._), 133.74 (CH), 144.86 (*C*_quat._), 145.11 (*C*_quat._), 159.13 (*C*_quat._), 167.73 (*C*_quat._).

#### 4.3.9 General Procedure for the Demethylation of RYR‐Stabilizer Compounds

In a baked‐out flask, the respective RYR‐stabilizer was dissolved in DCM (4 mL/mmol) and the solution was cooled to 0°C. At this temperature, BBr_3_ (1 M in DCM; 3.0 eq) were added and the mixture was warmed up to RT and was then stirred overnight. The reaction mixture was placed on ice and was then stirred for additional 30 min. Afterward, the phases were separated and the aqueous phase was extracted with EtOAc (3x). The combined organic phases were dried over MgSO_4_ and then the solvent was removed in vacuo. The resulting crude product was purified using a Chromabond C18 SPE cartridge (6cc) that was eluted with ACN/H_2_O. The gradient of elution was adjusted depending on the product of interest. The final products were the dried under a N_2_ stream.

#### 4.3.10 2,3,4,5‐Tetrahydrobenzo[*f*][1,4]thiazepine‐7‐ol (S107‐M2)

Isolated as yellow oil with a yield of 64.6%.

^1^H NMR (DMSO‐d_6_; 500 MHz): *δ* 2.62 (t; *J *= 4.8 Hz, 2H), 3.20 (t; *J *= 4.8 Hz, 2H), 3.88 (s, 2H), 6.57 (dd; *J *= 2.8, 8.2 Hz, 1H), 6.72 (d; *J *= 2.7 Hz, 1H), 7.31 (d; *J *= 8.2 Hz, 1H).

^13^C NMR (DMSO‐d_6_; 125 MHz): *δ* 36.48 (CH_2_), 53.72 (CH_2_), 55.39 (CH_2_), 113.74 (CH), 116.99 (CH), 125.26 (*C*_quat._), 133.85 (CH), 149.53 (*C*_quat._), 157.50 (*C*_quat._).

#### 4.3.11 4‐Methyl‐2,3,4,5‐tetrahydrobenzo[*f*][1,4]thiazepine‐7‐ol (S107‐M1b)

Isolated as yellow oil with a yield of 73.8%.

^1^H NMR (DMSO‐d_6_; 500 MHz): *δ* 2.82 (s, 3H), 3.16 (m; 2H), 3.60 (m; 2H), 4.52 (s, 2H), 6.80 (dd; *J *= 2.6, 8.3 Hz, 1H), 7.08 (d; *J *= 2.5 Hz, 1H), 7.38 (d; *J *= 8.4 Hz, 1H).

^13^C NMR (DMSO‐d_6_; 125 MHz): *δ* 36.94 (CH_2_), 53.32 (CH_3_), 58.84 (CH_2_), 59.43 (CH_2_), 117.08 (CH), 120.66 (CH), 126.38 (*C*_quat._), 134.35 (CH), 135.99 (*C*_quat._), 158.15 (*C*_quat._).

#### 4.3.12 3‐(4‐Benzylpiperidin‐1‐yl)‐1‐(7‐hydroxy‐2,3‐dihydrobenzo[*f*][1,4]thiazepin‐4(5H)‐yl)propan‐1‐one (JTV‐519‐M4)

Isolated as a colorless oil in quant. yields.

^1^H NMR (DMSO‐d_6_; 500 MHz): *δ* 1.47 (m, 2H), 1.71 (m, 2H), 2.53 (m, 1H), 2.69 (m, 1H), 2.82 (m, 4H), 2.92 (m, 1H), 3.19 (m, 3H), 3.41 (m, 2H), 3.91 (s, 2H), 4.51 (s, 1H), 4.66 (s, 1H), 6.58 (dd; *J *= 2.7, 8.3 Hz, 1H), 6.87 (d; *J *= 2.6 Hz, 1H), 7.24 (m, 6H).

^13^C NMR (DMSO‐d_6_; 125 MHz): *δ* 29.15 (CH_2_), 33.66 (CH_2_), 34.87 (CH_2_), 35.37 (CH), 41.86 (CH_2_), 52.12 (CH_2_), 52.61 (CH_2_), 53.06 (CH_2_), 114.66 (*C*_quat._), 119.08 (*C*_quat._), 124.30 (CH), 124.50 (CH), 126.53 (*C*_quat._), 128.76 (*C*_quat._), 129.48 (*C*_quat._), 134.07 (*C*_quat._), 139.90 (CH), 143.23 (CH), 143.97 (CH), 157.41 (CH).

#### 4.3.13 4‐((7‐Hydroxy‐2,3‐dihydrobenzo[*f*][1,4]thiazepin‐4(5H)‐yl)methyl)benzoic Acid (ARM 210‐M1)

Isolated as colorless solid with a yield of 81.5%.

^1^H NMR (DMSO‐d_6_; 500 MHz): *δ* 2.70 (m, 2H), 3.20 (m, 2H), 3.57 (s, 2H), 3.94 (s, 2H), 6.46 (m, 1H), 6.59 (d; *J *= 7.1 Hz, 1H), 7.30 (d; *J *= 8.1 Hz, 1H), 7.40 (d; *J *= 6.8 Hz, 2H), 7.90 (d; *J *= 7.1 Hz, 2H).

^13^C NMR (DMSO‐d_6_; 125 MHz): *δ* 30.47 (CH_2_), 55.85 (CH_2_), 58.55 (CH_2_), 59.47 (CH_2_), 114.65 (CH), 118.57 (CH), 125.68 (*C*_quat._), 129.02 (CH), 129.83 (CH), 129.97 (*C*_quat._), 133.81 (CH), 144.93 (*C*_quat._), 145.07 (*C*_quat._), 157.39 (*C*_quat._), 167.80 (*C*_quat._).

#### 4.3.14 2‐(7‐Hydroxy‐2,3‐dihydrobenzo[*f*][1,4]thiazepin‐4(5H)‐yl)‐2‐oxoacetic Acid (ARM 036‐M1)

The demethylated core structure (**S107‐M2**) (40 mg; 0.2 mmol; 1.0 eq) was diluted in DCM (2 mL; 10 mL/mmol) ethyl chloro oxoacetate (38 µL; 0.3 mmol; 1.5 eq) and pyridine (37 µL; 0.5 mmol; 2.0 eq) was added and the mixture was stirred for 1 h at RT. Then, the reaction was stopped by the addition of water, and both phases were separated. The aqueous phase was extracted with EtOAc (3x), and the combined organic phases were dried over MgSO_4_. After removing the solvent under reduced pressure, the intermediate product was dissolved in MeOH (3 mL; 10 mL/mmol) and NaOH (13 mg; 0.3 mmol; 1.0 eq) in H_2_O (3 mL; 10 mL/mmol) was added slowly. After 1.5 h, the reaction was stopped and extracted with EtOAc (3x). The combined organic phases were dried using MgSO_4_, and afterward, the solvent was removed in vacuo. The crude product was then purified using a Chromabond C18 SPE cartridge (6cc) that was eluted with ACN/H_2_O (10% to 75% ACN). After drying under a N_2_ stream, the final product was isolated as a white solid with a yield of 57 mg (64.2%).

^1^H NMR (DMSO‐d_6_; 500 MHz): *δ* 2.72 (t; *J *= 4.6 Hz, 2H), 3.76 (s (broad); 2H), 4.40 (s, 2H), 6.57 (dd; *J *= 2.6, 8.2 Hz, 1H), 6.87 (d; *J *= 2.5 Hz, 1H), 7.28 (d; *J *= 8.3 Hz, 1H), 9.74 (s; 1H).

^13^C NMR (DMSO‐d_6_; 125 MHz): *δ* 35.53 (CH_2_), 50.01 (CH_2_), 53.25 (CH_2_), 114.39 (CH), 118.90 (CH), 124.66 (*C*_quat._), 134.04 (CH), 144.81 (*C*_quat._), 157.45 (*C*_quat._), 167.54 (*C*_quat._), 170.27 (*C*_quat._).

### 4.4 In Vitro Metabolic Assay

For in vitro incubation, a protocol slightly divided from the protocol described by Kuuranne et al. was used [21]. HLMs were used to perform both phase‐I and phase‐II metabolisms, and a NADPH regenerating system (NADPH reg system) was used for NADPH supply during the experiment. Each RYR‐stabilizer was diluted in MeOH to produce a stock solution with a concentration of 2 mg/mL. Afterward, the stock solutions were diluted in 50 mM phosphate buffer (pH 7.4) containing 5 mM MgCl_2_ to produce a working solution (200 µM; resulting concentrations for each compound: S107 = 41.9 µg/mL; JTV‐519 = 84.9 µg/mL; ARM 036 = 53.5 µg/mL; ARM 210 = 65.9 µg/mL). For phase‐I metabolism, 10 µL working solution, 10 µL NADPH reg system (50 mM), and 5 µL HLM (20 mg/mL) were added in 25 µL phosphate buffer for a total volume of 50 µL. All samples were incubated at 37°C for 24  h. For phase‐II metabolism, additional 5 µL of HLM and 10 µL of UDGPA (50 mM) and 10 µL of SL (50 mM) were added, and the mixture was again incubated at 37°C for 24 h. Together with the in vitro samples, blanks either excluding HLMs or excluding the respective substrate were performed to verify the results obtained in this study. Following incubation, each metabolism step was quenched by the addition of 150 µL ice‐cold ACN. The supernatant was harvested after centrifugation (17,000 × g, 5 min) and transferred into a fresh tube. After drying using a vacuum centrifuge (45°C, 45 min), the samples were reconstituted in 100 µL ACN:H_2_O (90:10 v/v) and diluted by a factor of 1:20, again using ACN:H_2_O (90:10 v/v).

#### 4.4.1 LC‐HRMS

LC‐MS measurements were performed using a Vanquish UHPLC system coupled to an Orbitrap Exploris 480 mass spectrometer (Thermo Fisher (Bremen, Germany)). The HPLC system was equipped with an EC 4/3 Nucleoshell RP 18 Plus guard column (4 × 3 mm, 5 μm particle size; Macherey–Nagel, Düren, Germany) and a Poroshell 120 EC C18 column (3.0 × 50 mm, 2.7 µm) by Agilent (Santa Clara, California, USA). For gradient elution, 0.1% FA in H_2_O was used as eluent A and 0.1% FA in ACN was used as eluent B. The gradient started at 0% B and was then increased to 50% B within 10 min, and over an additional 2 min, the gradient was brought to 100% B where it was held for 1 min. After returning to starting conditions within 0.01 min, the column was re‐equilibrated for 2 min. A flow of 0.3 mL/min was applied. The injection volume was 10 µL.

In the MS system a heated ESI source with a voltage of 3000 V was used for ionization in positive mode. Both full scan data and product ion scans were monitored. In full scan mode, the orbitrap was used with a resolution of 60,000 full width at half maximum (FWHM) and at a scan range of 50–800 *m*/*z*. Product ion scans were generated using parallel reaction monitoring also in positive ionization mode, using a resolution of 30,000 FWHM. The isolation window was set to 1.3 *m*/*z*. For higher energy collisional dissociation, normalized collision energies between 25% and 55% were used, depending on the *m*/*z* ratio of interest. In addition, pseudo MS^3^ experiments were conducted, using in‐source fragmentation. Nitrogen was used as collision gas and generated by a CMC nitrogen generator (Eschborn, Germany). The MS was regularly calibrated using the Pierce Flex Mix calibration solution from Thermo Fisher (Bremen, Germany).

### 4.5 Acetylation

For acetylation, 10 µL of the undiluted in vitro sample was dried using a nitrogen stream. Afterward, 500 µL of Ac_2_O and pyridine was added. The mixture was incubated at 50°C for 1 h before the solvent and reactants were removed via a nitrogen stream, and the residue was reconstituted in 100 µL of ACN:H_2_O (90:10 v/v).

## Supporting Information

Additional supporting information can be found online in the Supporting Information Section. **Supporting Fig. S1**: ^1^H‐NMR of **2a**. **Supporting Fig. S2**: ^13^C‐NMR of **2a**. **Supporting Fig. S3**: ^1^H‐NMR of **3a**. **Supporting Fig. S4**: ^13^C‐NMR of **3a**. **Supporting Fig. S5**: ^1^H‐NMR of **4a**. **Supporting Fig. S6**: ^13^C‐NMR of **4a**. **Supporting Fig. S7**: ^1^H‐NMR of **5a**. **Supporting Fig. S8**: ^13^C‐NMR of **5a**. **Supporting Fig. S9**: ^1^H‐NMR of **S107**. **Supporting Fig. S10**: ^13^C‐NMR of **S107**. **Supporting Fig. S11**: ^1^H‐NMR of **JTV‐519**. **Supporting Fig. S12**: ^13^C‐NMR of **JTV‐519**. **Supporting Fig. S13**: ^1^H‐NMR of **ARM 036**. **Supporting Fig. S14**: ^13^C‐NMR of **ARM 036**. **Supporting Fig. S15**: ^1^H‐NMR of **ARM 210**. **Supporting Fig. S16**: ^13^C‐NMR of **ARM 210**. **Supporting Fig. S17**: ^1^H‐NMR of **S107‐M2**. **Supporting Fig. S18**: ^13^C‐NMR of **S107‐M2**. **Supporting Fig. S19**: ^1^H‐NMR of **S107‐M1b**. **Supporting Fig. S20**: ^13^C‐NMR of **S107‐M1b**. **Supporting Fig. S21**: ^1^H‐NMR of **JTV‐519‐M4**. **Supporting Fig. S22**: ^13^C‐NMR of **JTV 519‐M4**. **Supporting Fig. S23**: ^1^H‐NMR of **ARM 036‐M1**. **Supporting Fig. S24**: ^13^C‐NMR of **ARM 036‐M1**. **Supporting Fig. S25**: ^1^H‐NMR of **ARM 210‐M1**. **Supporting Fig. S26**: ^13^C‐NMR of **ARM 210‐M1**. **Supporting Table S1**: List of in‐vitro metabolites detected for S107, including the product ions obtained for each metabolite. The extraction window for MS^2^ experiments was set at *m/z* = 1.3 and product ions wthin an error of 5 ppm were accepted. **Supporting Table S2**: List of in‐vitro metabolites detected for JTV‐519, including the product ions obtained for each metabolite. The extraction window for MS^2^ experiments was set at *m/z* = 1.3 and product ions wthin an error of 5 ppm were accepted. **Supporting Table S3**: List of in‐vitro metabolites detected for ARM 036, including the product ions obtained for each metabolite. The extraction window for MS^2^ experiments was set at *m/z* = 1.3 and product ions wthin an error of 5 ppm were accepted. **Supporting Table S4**: List of in‐vitro metabolites detected for ARM 210, including the product ions obtained for each metabolite. The extraction window for MS^2^ experiments was set at *m/z* = 1.3 and product ions wthin an error of 5 ppm were accepted.

## Funding

This study was supported by World Anti‐Doping Agency (Grant #241C07MT), Ingeborg Gross Foundation, Manfred Donike Institut für Dopinganalytik, and Bundesministerium des Innern, für Bau und Heimat.

## Conflicts of Interest

The authors declare no conflicts of interest.

## Supporting information

Supplementary Material

## Data Availability

The data that support the findings of this study are available in the Supporting Information of this article.
