# Supplementary material for: Ryanodine‐1‐Calstabin Complex Stabilizers in Antidoping Research: Synthesis, Metabolism, and Characterization
Source: Chempluschem. 2025 Dec 27;91(1):e202500493. doi: 10.1002/cplu.202500493 (PMC12743319; doi:10.1002/cplu.202500493)
Supplement: Supplementary file 1 — Supplementary Material [file CPLU-91-e202500493-s001.pdf]

Supporting information

Ryanodine-1-Calstabin Complex Stabilizers in Anti-Doping Research: Synthesis, Metabolism, and Characterization

Tristan Möller[a], Thomas Piper[a], Mario Thevis*[a][b]

Table 1: List of in-vitro metabolites detected for S107, including the product ions obtained for each metabolite. The extraction window for MS^2 experiments was set at m/z = 1.3 and product ions wthin an error of 5 ppm were accepted........ 2
Table 2: List of in-vitro metabolites detected for JTV-519, including the product ions obtained for each metabolite. The extraction window for MS^2 experiments was set at m/z = 1.3 and product ions wthin an error of 5 ppm were accepted. 3
Table 3: List of in-vitro metabolites detected for ARM 036, including the product ions obtained for each metabolite. The extraction window for MS^2 experiments was set at m/z = 1.3 and product ions wthin an error of 5 ppm were accepted. 4
Table 4: List of in-vitro metabolites detected for ARM 210, including the product ions obtained for each metabolite. The extraction window for MS^2 experiments was set at m/z = 1.3 and product ions wthin an error of 5 ppm were accepted. 4

Figure 1: ^{1}H-NMR of **2a**........ 5
Figure 2: ^{13}C-NMR of **2a**........ 5
Figure 3: ^{1}H-NMR of **3a**........ 6
Figure 4: ^{13}C-NMR of **3a**........ 6
Figure 5: ^{1}H-NMR of **4a**........ 7
Figure 6: ^{13}C-NMR of **4a**........ 7
Figure 7: ^{1}H-NMR of **5a**........ 8
Figure 8: ^{13}C-NMR of **5a**........ 8
Figure 9: ^{1}H-NMR of **S107**........ 9
Figure 10: ^{13}C-NMR of **S107**........ 9
Figure 11^{1}H-NMR of **JTV-519**........ 10
Figure 12: ^{13}C-NMR of **JTV-519**........ 10
Figure 13: ^{1}H-NMR of **ARM 036**. 11
Figure 14: ^{13}C-NMR of **ARM 036**........ 11
Figure 15: ^{1}H-NMR of **ARM 210**. 12
Figure 16: ^{13}C-NMR of **ARM 210**........ 12
Figure 17: ^{1}H-NMR of **S107-M2**........ 13
Figure 18: ^{13}C-NMR of **S107-M2**. 13
Figure 19^{1}H-NMR of **S107-M1b**........ 14
Figure 20: 13C-NMR of **S107-M1b**. 14
Figure 21: ^{1}H-NMR of **JTV-519-M4**........ 15
Figure 22 : ^{13}C-NMR of **JTV 519-M4.** 15
Figure 23: ^{1}H-NMR of **ARM 036-M1**........ 16
Figure 24: ^{13}C-NMR of **ARM 036-M1**........ 16
Figure 25: ^{1}H-NMR of **ARM 210-M1**........ 17
Figure 26: ^{13}C-NMR of **ARM 210-M1**........ 17

Table 1: List of *in-vitro* metabolites detected for S107, including the product ions obtained for each metabolite. The extraction window for MS^2 experiments was set at m/z = 1.3 and product ions wthin an error of 5 ppm were accepted.

compound	transformation	parent ion [*m/z*]	formula	retention time [min]	product ions [m/z]	predicted formula
**S107**		210.0947	$C_{11}H_{16}NOS^+$	5.88	179.0525	$C_{10}H_{11}OS^+$
					153.0374	$C_8H_9OS^+$
					138.0134	$C_7H_6OS^+$
					107.0489	$C_7H_7O^+$
**S107-M1a**	demethylation	196.0796	$C_{10}H_{14}NOS^+$	4.64	167.0525	$C_9H_{11}OS^+$
					165.0369	$C_9H_9OS^+$
					139.0218	$C_7H_7OS^+$
**S107-M1b**	demethylation	196.0796	$C_{10}H_{14}NOS^+$	5.78	179.0525	$C_{10}H_{11}OS^+$
					153.0374	$C_8H_9OS^+$
					138.0134	$C_7H_6OS^+$
**S107-M2**	di-demethylation	182.0634	$C_9H_{12}NOS^+$	4.59	165.0369	$C_9H_9OS^+$
					150.0139	$C_8H_6OS^+$
					139.0218	$C_7H_7OS^+$
**S107-M3a**	*C* oxidation	224.0740	$C_{11}H_{14}NO_2S^+$	8.52	193.0311	$C_{10}H_9O_2S^+$
					165.0363	$C_9H_9OS^+$
					153.0363	$C_8H_9OS^+$
**S107-M3b**	*C* oxidation	224.0740	$C_{11}H_{14}NO_2S^+$	9.28	196.0785	$C_{10}H_{14}NOS^+$
					179.0525	$C_{10}H_{11}OS^+$
					153.0363	$C_8H_9OS^+$
**S107-M3c**	*C* oxidation	224.0740	$C_{11}H_{14}NO_2S^+$	9.42	196.0791	$C_{10}H_{14}NOS^+$
					179.0525	$C_{10}H_{11}OS^+$
					153.0374	$C_8H_9OS^+$
**S107-M4a**	*S* oxidation	226.0896	$C_{11}H_{16}NO_2S^+$	3.50	208.0791	$C_{11}H_{14}NOS^+$
					179.0525	$C_{10}H_{11}OS^+$
					166.0452	$C_9H_{10}OS^+$
					153.0374	$C_8H_9OS^+$
**S107-M4b**	hydroxylation	226.0896	$C_{11}H_{16}NO_2S^+$	4.13	208.0791	$C_{11}H_{14}NOS^+$
					165.0374	$C_9H_9OS^+$
					153.0374	$C_8H_9OS^+$
					139.0218	$C_7H_7OS^+$
**S107-M4c**	*N* oxidation	226.0896	$C_{11}H_{16}NO_2S^+$	6.15	181.0556	$C_9H_{11}NOS^{\bullet+}$
					154.0447	$C_8H_{10}OS^{\bullet+}$
					153.0374	$C_8H_9OS^{\bullet+}$
**S107-M5**	hydroxylation + *S* oxidation			5.24	211.0425	$C_{10}H_{11}O_3S^+$
					193.0319	$C_{10}H_9O_2S^+$
					183.0474	$C_9H_{11}O_2S^+$
					165.0369	$C_9H_9OS^+$
**S107-M1b-*O*-Gluc**	demethylation + glucuronidation	372.1111	$C_{16}H_{22}NO_7S^+$	3.67	196.0791	$C_{10}H_{14}NOS^+$
					167.0525	$C_9H_{11}OS^+$
					165.0369	$C_9H_9OS^+$
					139.0218	$C_7H_7OS^+$
**S107-M1b-*N*-Gluc**	demethylation + glucuronidation	372.1111	$C_{16}H_{22}NO_7S^+$	4.40	196.0791	$C_{10}H_{14}NOS^+$
					159.0288	$C_6H_7O_5^+$
					131.0339	$C_5H_7O_4^+$
					113.0233	$C_5H_5O_3^+$
**S107-M2-*O*-Gluc**	di-demethylation + glucuronidation	358.0955	$C_{15}H_{20}NO_7S^+$	3.73	182.0634	$C_9H_{12}NOS^+$
					165.0369	$C_9H_9OS^+$
					139.0218	$C_7H_7OS^+$
					113.0233	$C_5H_5O_3^+$
**S107-*O*-Gluc-1**	glucuronidation	386.1286	$C_{17}H_{24}NO_7S^+$	5.20	210.0947	$C_{11}H_{16}NOS^+$
					179.0525	$C_{10}H_{11}OS^+$
					153.0374	$C_8H_9OS^+$
					113.0233	$C_5H_5O_3^+$
**S107-*O*-Gluc-2**	glucuronidation	386.1286	$C_{17}H_{24}NO_7S^+$	5.51	210.0947	$C_{11}H_{16}NOS^+$
					179.0525	$C_{10}H_{11}OS^+$
					153.0374	$C_8H_9OS^+$
					113.0233	$C_5H_5O_3^+$

Table 2: List of *in-vitro* metabolites detected for JTV-519, including the product ions obtained for each metabolite. The extraction window for MS^2 experiments was set at *m/z* = 1.3 and product ions wthin an error of 5 ppm were accepted.

compound	transformation	parent mass [m/z]	formula	retention time [min]	product ions [m/z]	predicted formula
**JTV-519**		425.2257	$C_{25}H_{33}N_2O_2S^+$	10.54	188.1434	$C_{13}H_{18}N^+$
**JTV-519-M1**	amid cleavage	248.1651	$C_{15}H22NO2^+$	6.78	188.1440	$C_{13}H_{18}N^+$
**JTV-519-M2**	amin cleavage	176.1434	$C_{12}H_{18}N^+$	6.58	117.0702<br>91.0545	$C_{12}H_{18}N^+$<br>$C_7H_7^+$
**JTV-519-M3**	amin cleavage + oxidation	192.139	$C_{12}H_{18}NO^+$	4.63	133.0653<br>107.0494<br>98.0967	$C_9H_9O^+$<br>$C_7H_7O^+$<br>$C_6H_{12}N^+$
**JTV-519-M4**	demethylation	411.2101	$C_{24}H_{31}N_2O_2S^+$	9.28	188.1434<br>164.0528	$C_9H_{10}NS^+$<br>$C_{13}H_{18}N^+$
**JTV-519-M5a**	demethylation + oxidation	427.2059	$C_{24}H_{31}N_2O_3S^+$	7.47	252.0689<br>188.1440<br>180.0478<br>139.0212	$C_{12}H_{14}NO_3S^+$<br>$C_{13}H_{18}N^+$<br>$C_9H_{10}NOS^+$<br>$C_7H_7OS^+$
**JTV-519-M5b**	demethylation + oxidation	427.2059	$C_{24}H_{31}N_2O_3S^+$	7.88	204.1390	$C_{13}H_{18}NO^+$
**JTV-519-M5c**	demethylation + oxidation	427.2059	$C_{24}H_{31}N_2O_3S^+$	8.83	188.1441	$C_{13}H_{18}N^+$
**JTV-519-M6a**	demethylation + bis oxidation	443.2014	$C_{24}H_{31}N_2O_4S^+$	5.88	252.0689<br>204.1390<br>180.0478<br>139.0212	$C_{12}H_{14}NO_3S^+$<br>$C_{13}H_{18}NO^+$<br>$C_9H_{10}NOS^+$<br>$C_7H_7OS^+$
**JTV-519-M6b**	demethylation + bis oxidation	443.2014	$C_{24}H_{31}N_2O_4S^+$	7.14	196.0434<br>188.1441	$C_9H_{10}NO_2S^+$<br>$C_{13}H_{18}N^+$
**JTV-519-M6c**	demethylation + bis oxidation	443.2014	$C_{24}H_{31}N_2O_4S^+$	7.33	220.1339	$C_{13}H_{18}NO2^+$
**JTV-519-M6d**	demethylation + bis oxidation	443.2014	$C_{24}H_{31}N_2O_4S^+$	8.2	268.0638<br>196.0427<br>188.1434	$C_{12}H_{14}NO_4S^+$<br>$C_9H_{10}NO_2S^+$<br>$C_{13}H_{18}N^+$
**JTV-519-M7a**	oxidation	441.2206	$C_{11}H_{14}NO_2S^+$	8.36	423.2101<br>266.0845<br>230.1539<br>194.0634<br>188.1434<br>153.0374	$C_{25}H_{31}N_2O_2S^+$<br>$C_{13}H_{16}NO_3S^+$<br>$C_{15}H_{20}NO^+$<br>$C_{10}H_{12}NOS^+$<br>$C_{13}H_{18}N^+$<br>$C_8H_9OS^+$
**JTV-519-M7b**	oxidation	441.2206	$C_{11}H_{14}NO_2S^+$	9.07	423.2101<br>204.1383<br>188.1434	$C_{25}H_{31}N_2O_2S^+$<br>$C_{13}H_{18}NO^+$<br>$C_{13}H_{18}N^+$
**JTV-519-M8a**	bis oxidation	457.2156	$C_{25}H_{33}N_2O_4S^+$	6.82	439.2050<br>266.0845<br>204.1383<br>194.0634	$C_{25}H_{31}N_2O_3S^+$<br>$C_{13}H_{16}NO_3S^+$<br>$C_{13}H_{18}NO^+$<br>$C_{10}H_{12}NOS^+$
**JTV-519-M8b**	bis oxidation	457.2156	$C_{25}H_{33}N_2O_4S^+$	7.06	439.2050<br>266.0845<br>204.1383<br>194.0634	$C_{25}H_{31}N_2O_3S^+$<br>$C_{13}H_{16}NO_3S^+$<br>$C_{13}H_{18}NO^+$<br>$C_{10}H_{12}NOS^+$
**JTV-519-M8c**	bis oxidation	457.2156	$C_{25}H_{33}N_2O_4S^+$	7.78	220.1332	$C_{13}H_{18}NO_2^+$
**JTV-519-M8d**	bis oxidation	457.2156	$C_{25}H_{33}N_2O_4S^+$	9.18	188.1434	$C_9H_{10}NS^+$
**JTV-519-M9a**	tri oxidation	473.2105	$C_{25}H_{33}N_2O_5S^+$	5.44	455.2013<br>347.1796<br>266.0853<br>220.1338<br>194.0640	$C_{25}H_{31}N_2O_4S^+$<br>$C_{19}H_{27}N_2O_2S^+$<br>$C_{13}H_{16}NO_3S^+$<br>$C_{13}H_{18}NO_2^+$<br>$C_{10}H_{12}NOS^+$
**JTV-519-M9b**	tri oxidation	473.2105	$C_{25}H_{33}N_2O_5S^+$	6.32	455.2013<br>266.0853<br>220.1338<br>194.0640	$C_{25}H_{31}N_2O_4S^+$<br>$C_{13}H_{16}NO_3S^+$<br>$C_{13}H_{18}NO_2^+$<br>$C_{10}H_{12}NOS^+$
**JTV-519-M9c**	tri oxidation	473.2105	$C_{25}H_{33}N_2O_5S^+$	7.65	455.2013<br>204.1383	$C_{25}H_{31}N_2O_4S^+$<br>$C_{13}H_{18}NO^+$
**JTV-519-M7a-*O*-Gluc-1**	oxidation + glucuronidation	617.2527	$C_{31}H_{41}N_2O_9S^+$	7.66	441.2191<br>188.1427	$C_{11}H_{14}NO_2S^+$<br>$C_9H_{10}NS^+$
**JTV-519-M7a-*O*-Gluc-2**	oxidation + glucuronidation	617.2527	$C_{31}H_{41}N_2O_9S^+$	7.94	441.2194<br>380.1693<br>204.1377	$C_{11}H_{14}NO_2S^+$<br>$C_{19}H_{26}NO_7^+$<br>$C_{13}H_{18}NO^+$
**JTV-519-M7a-*O*-Gluc-3**	oxidation + glucuronidation	617.2527	$C_{31}H_{41}N_2O_9S^+$	8.16	441.2193<br>380.1693<br>204.1376	$C_{11}H_{14}NO_2S^+$<br>$C_{19}H_{26}NO_7^+$<br>$C_{13}H_{18}NO^+$
**JTV-519-M7a-*O*-Gluc-4**	oxidation + glucuronidation	617.2527	$C_{31}H_{41}N_2O_9S^+$	8.56	441.2196<br>188.1429	$C_{11}H_{14}NO_2S^+$<br>$C_9H_{10}NS^+$

Table 2: List of *in-vitro* metabolites detected for JTV-519, including the product ions obtained for each metabolite. The extraction window for MS^2 experiments was set at *m/z* = 1.3 and product ions wthin an error of 5 ppm were accepted. (continued)

compound	transformation	parent mass [m/z]	formula	retention time [min]	product ions [m/z]	predicted formula
**JTV-519-M8a-*O*-Gluc-1**	bis oxidation + glucuronidation	633.2476	$C_{31}H_{41}N_2O_{10}S^+$	5.81	457.2140	$C_{25}H_{33}N_2O_4S^+$
					380.1688	$C_{19}H_{26}NO_7^+$
					204.1376	$C_{13}H_{18}NO^+$
**JTV-519-M8a-*O*-Gluc-2**	bis oxidation + glucuronidation	633.2476	$C_{31}H_{41}N_2O_{10}S^+$	6.38	457.2144	$C_{25}H_{33}N_2O_4S^+$
					204.1376	$C_{13}H_{18}NO^+$
**JTV-519-M8a-*O*-Gluc-3**	bis oxidation + glucuronidation	633.2476	$C_{31}H_{41}N_2O_{10}S^+$	6.78	457.2144	$C_{25}H_{33}N_2O_4S^+$
					210.0578	$C_{10}H_{12}NO_2S^+$
					188.1428	$C_9H_{10}NS^+$
**JTV-519-M8a-*O*-Gluc-4**	bis oxidation + glucuronidation	633.2476	$C_{31}H_{41}N_2O_{10}S^+$	7.84	457.2141	$C_{25}H_{33}N_2O_4S^+$
					396.1642	$C_{19}H_{26}NO_8^+$
					220.1327	$C_{13}H_{18}NO_2^+$

Table 3: List of *in-vitro* metabolites detected for ARM 036, including the product ions obtained for each metabolite. The extraction window for MS^2 experiments was set at *m/z* = 1.3 and product ions wthin an error of 5 ppm were accepted.

compound	transformation	parent ion [*m/z*]	formula	retention time [min]	product ions [m/z]	Predicted formula
**ARM 036**		268.0638	$C_{12}H_{13}NO_4S^+$	7.65	222.0578	$C_{11}H_{12}NO_2S^+$
					196.0785	C_{10}H14OS$^+$
					194.0629	$C_{10}H_{12}OS$+
					179.0521	$C_7H_6OS^+$
					153.0363	$C_8H_9OS^+$
					139.0208	$C_7H_7OS^+$
**ARM 036-M1**	demethylation	254.0791	$C_{11}H_{12}NO_4S^+$	6.17	208.0436	$C_{10}H_{10}NO_2S^+$
					165.0369	$C_9H_9OS^+$
					139.0218	$C_7H_7OS^+$
**ARM 036-M2**	oxidation	284.0587	$C_{12}H_{14}NO_5S^+$	4.54	240.0684	$C_{11}H_{14}NO_3S^+$
					212.0736	$C_{10}H_{14}NO_2S^+$
					194.0630	$C_{10}H_{12}NOS^+$
					177.0365	$C_{10}H_9OS^+$

Table 4: List of *in-vitro* metabolites detected for ARM 210, including the product ions obtained for each metabolite. The extraction window for MS^2 experiments was set at *m/z* = 1.3 and product ions wthin an error of 5 ppm were accepted.

compound	transformation	parent ion [*m/z*]	formula	retention time [min]	product ions [m/z]	predicted formula
**ARM 210**		330.1158	$C_{18}H_{20}NO_3S^+$	7.54	273.0574	$C_{15}H_{13}NO_3S^+$
					208.0420	$C_{10}H_{10}O_2S^+$
					167.0394	$C_9H_{11}OS$+
					153.0364	$C_8H_9OS^+$
					138.0130	$C_8H_9OS^+$
					135.0436	$C_8H_7O_2^+$
**ARM 210-M1**	demethylation	316.1002	$C_{17}H_{18}NO_3S^+$	6.27	176.0702	$C_{10}H_{10}NO_2^+$
					153.0239	$C_7H_7NOS^{\bullet+}$
					139.0208	$C_7H_7OS^+$
					135.0436	$C_8H_7O_2^+$
**ARM 210-M2**	oxidation	346.1108	$C_{10}H_{14}NOS^+$	6.04	328.0990	$C_{11}H_{14}NO_3S^+$
					183.0468	$C_9H_{11}O_2S^+$
					135.0436	$C_8H_7O_2^+$
**ARM 210-M1-*O*-Gluc**	demethylation + glucuronidation	492.1323	$C_{23}H_{26}NO_9S^+$	5.06	316.0992	$C_{17}H_{18}NO_3S^+$
					139.0208	$C_7H_7OS^+$
					135.0436	$C_8H_7O_2^+$

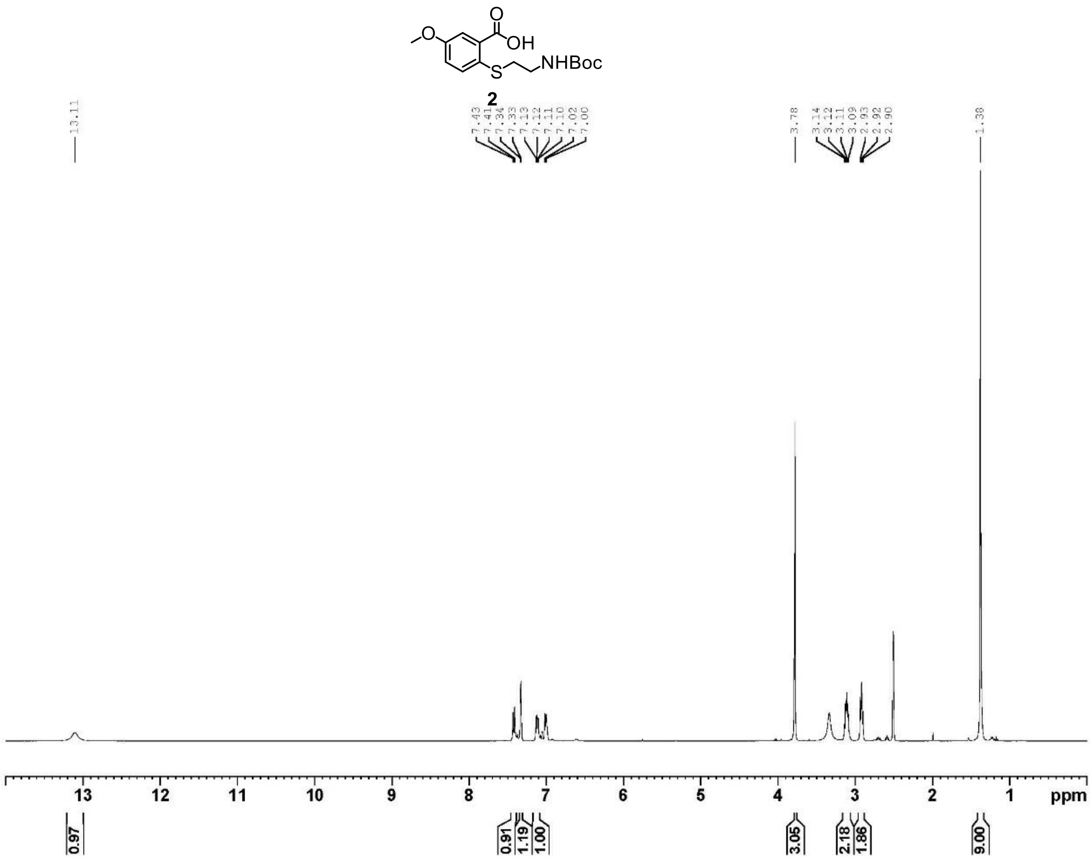

Figure 1: ^{1}H-NMR of **2a**.

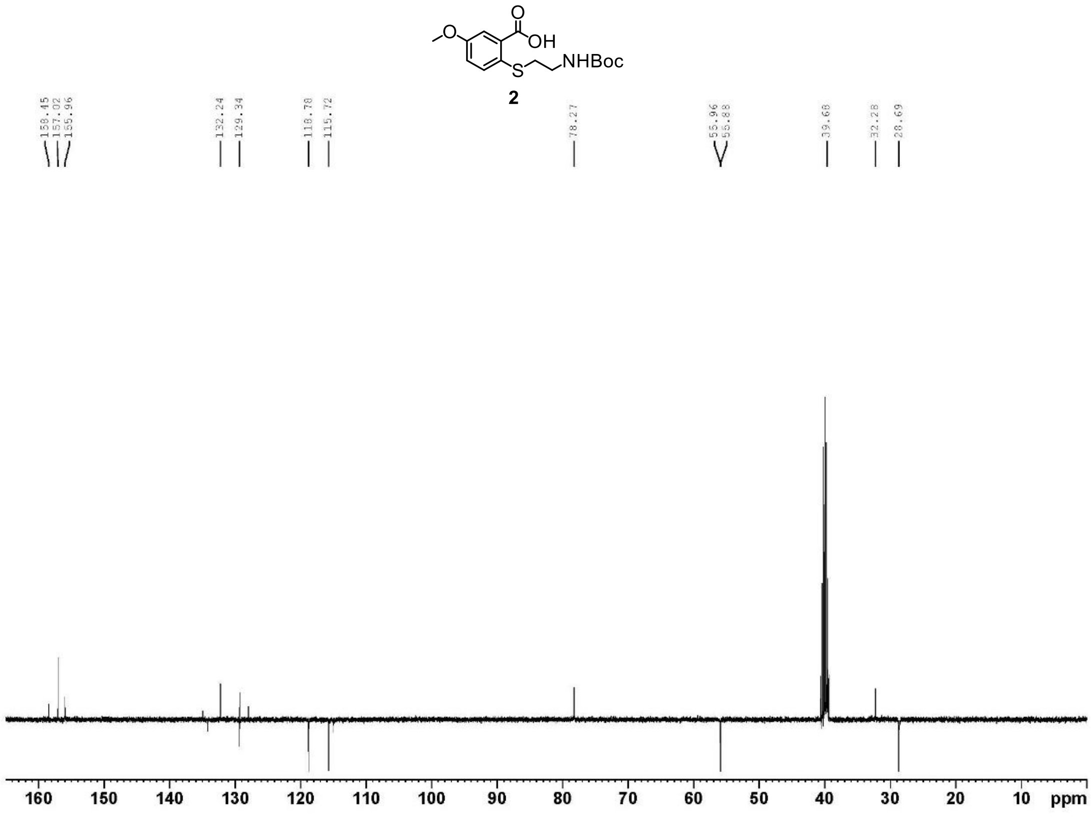

Figure 2: ^{13}C-NMR of **2a**.

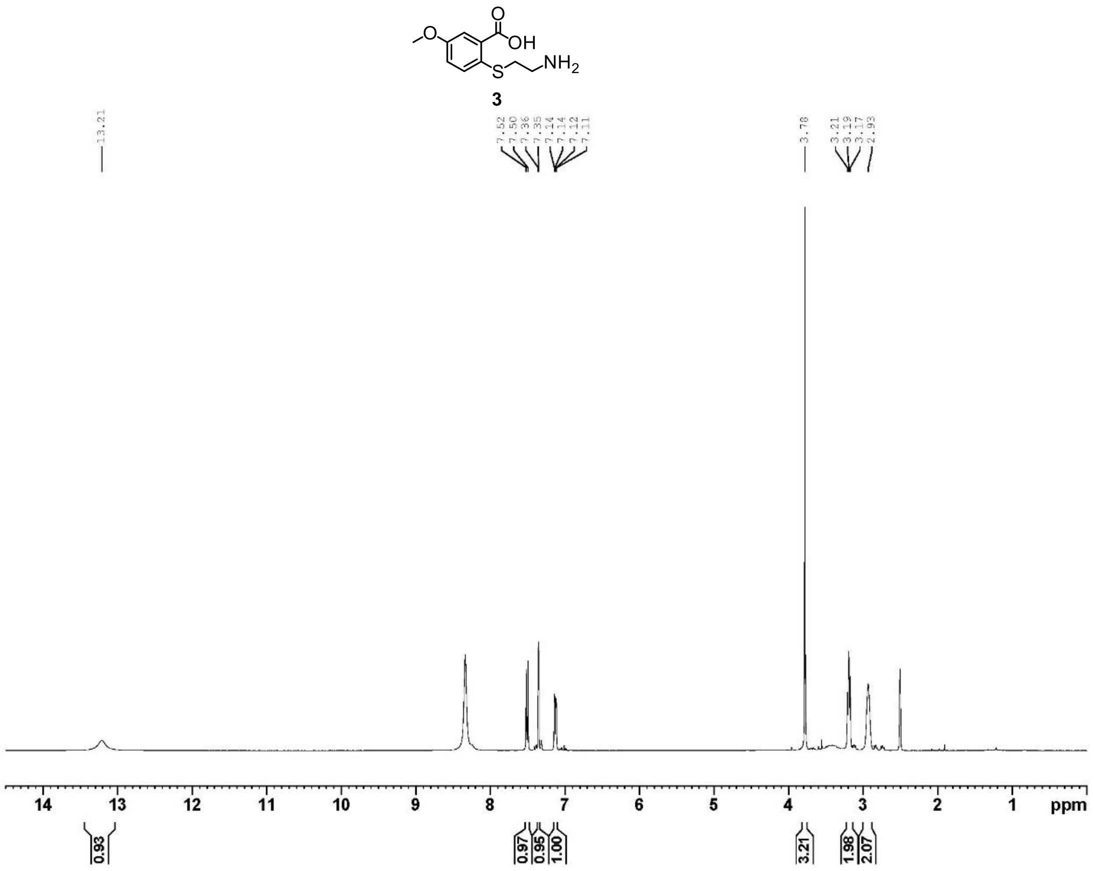

Figure 3: ^{1}H-NMR of **3a**.

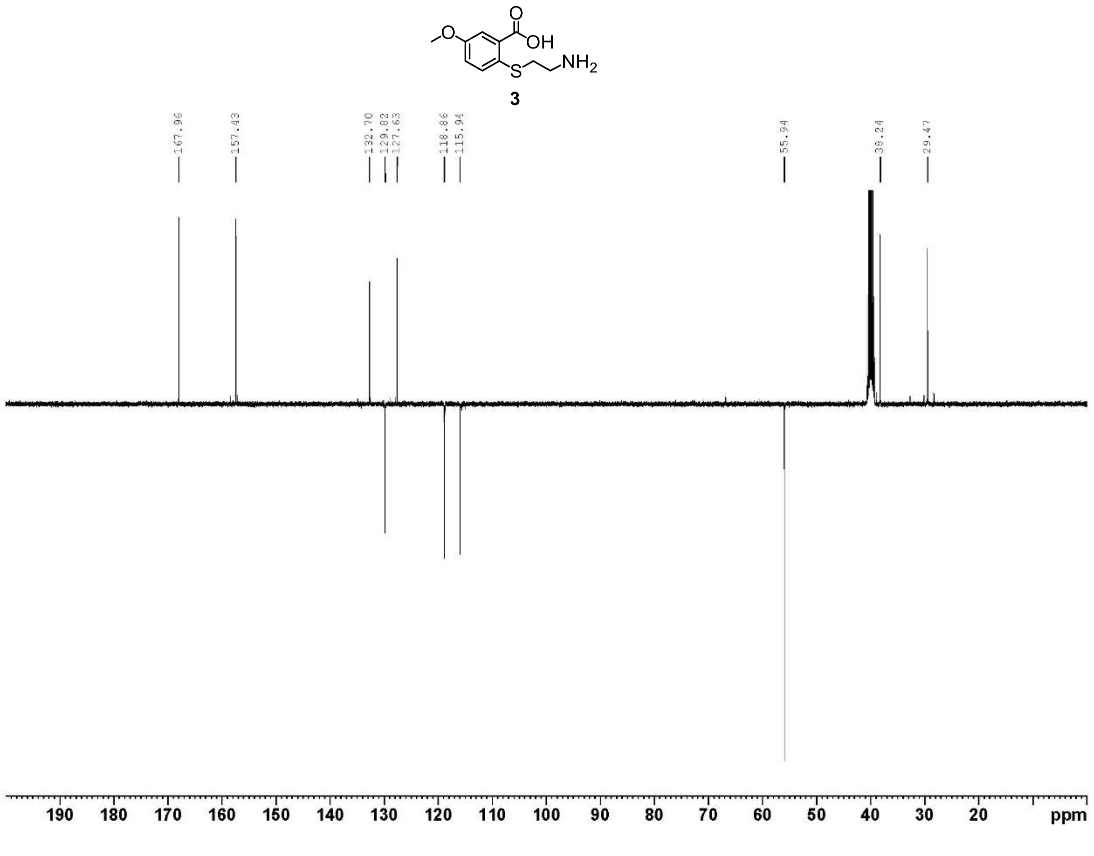

Figure 4: ^{13}C-NMR of **3a**.

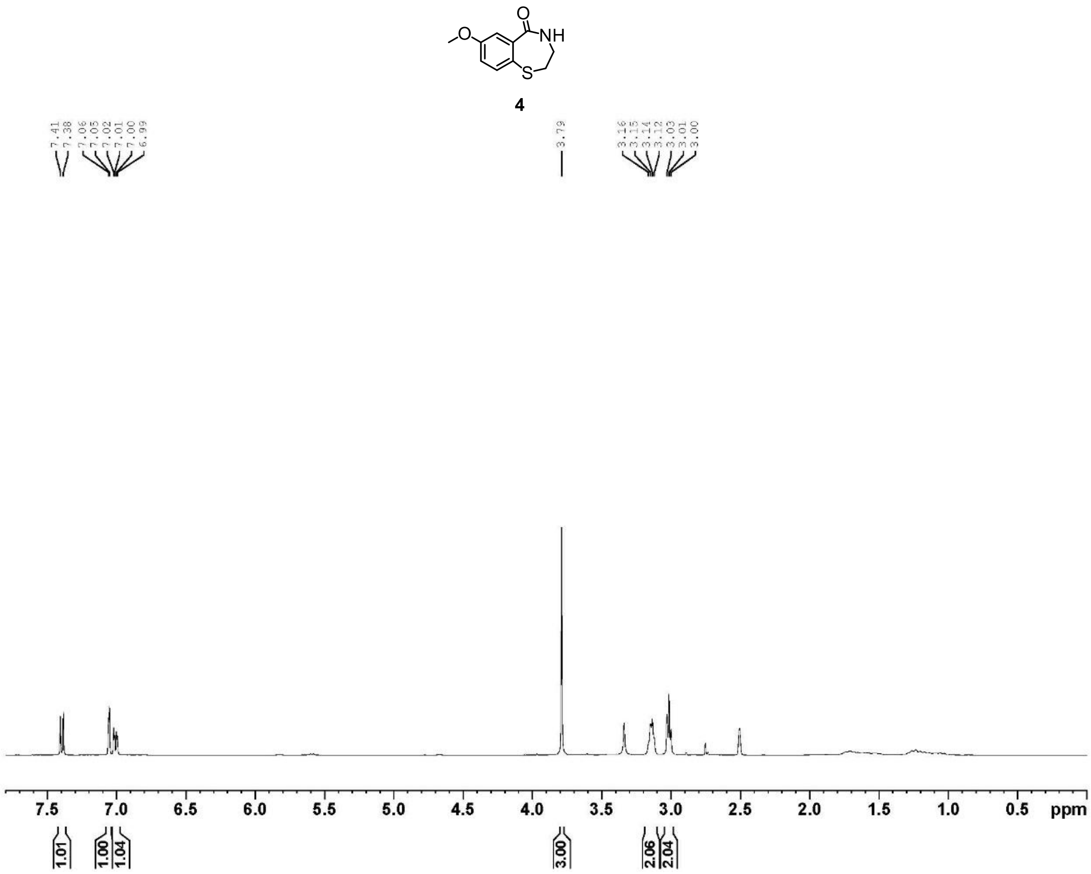

Figure 5: ^{1}H-NMR of **4a**.

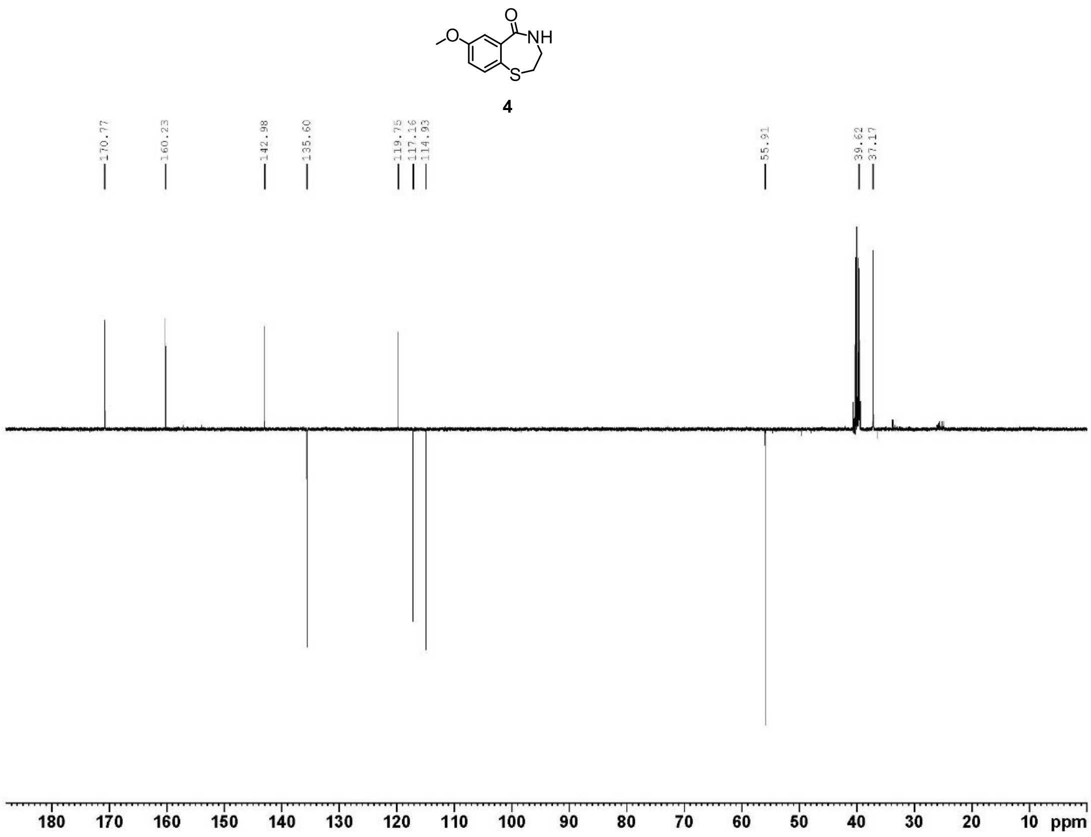

Figure 6: ^{13}C-NMR of **4a**.

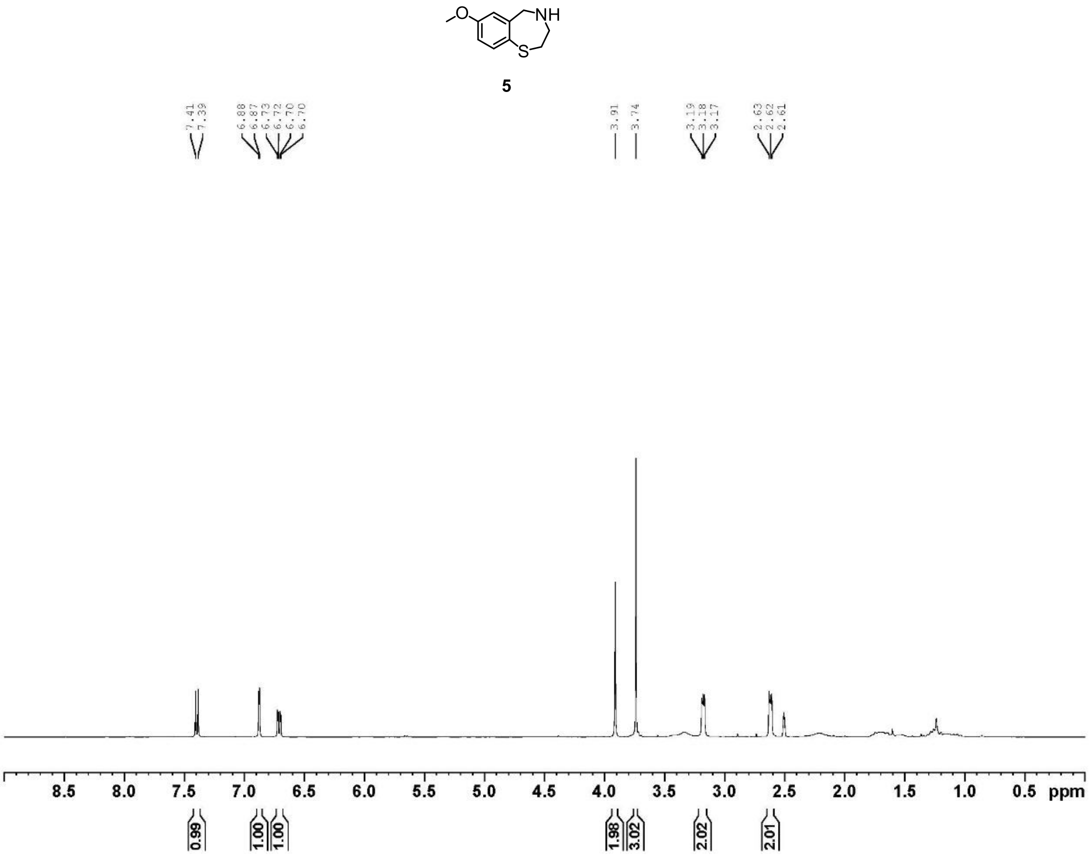

Figure 7: ^{1}H-NMR of **5a**.

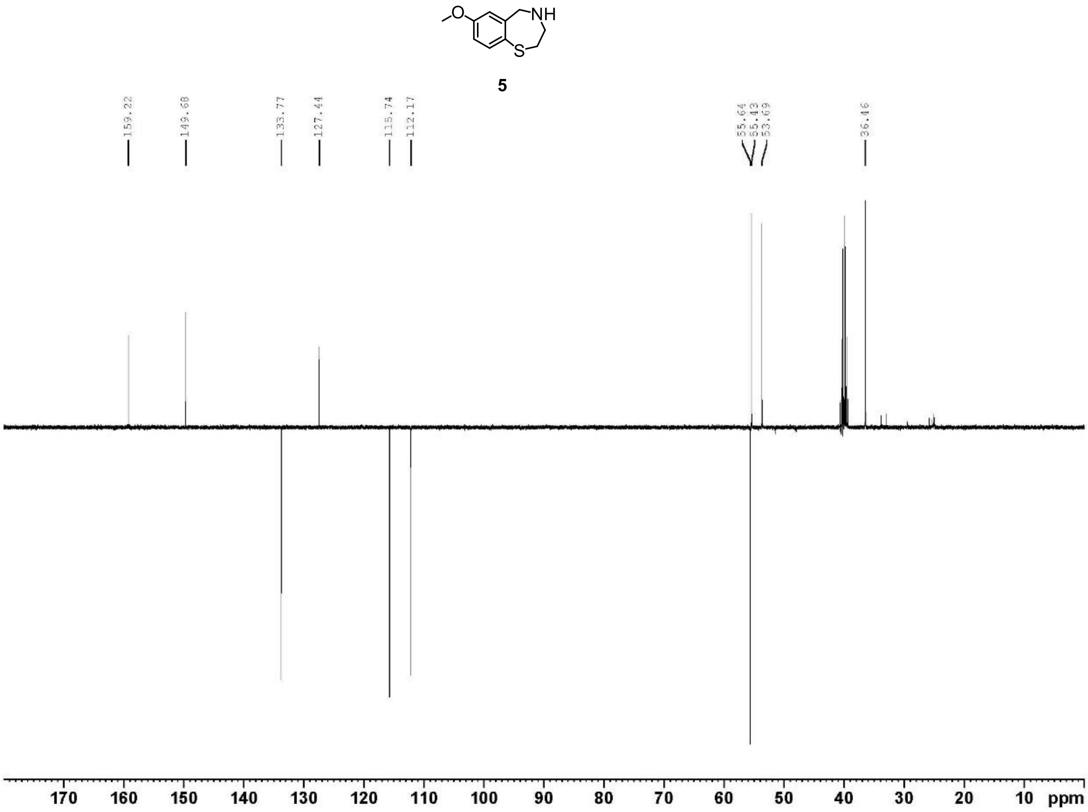

Figure 8: ^{13}C-NMR of **5a**.

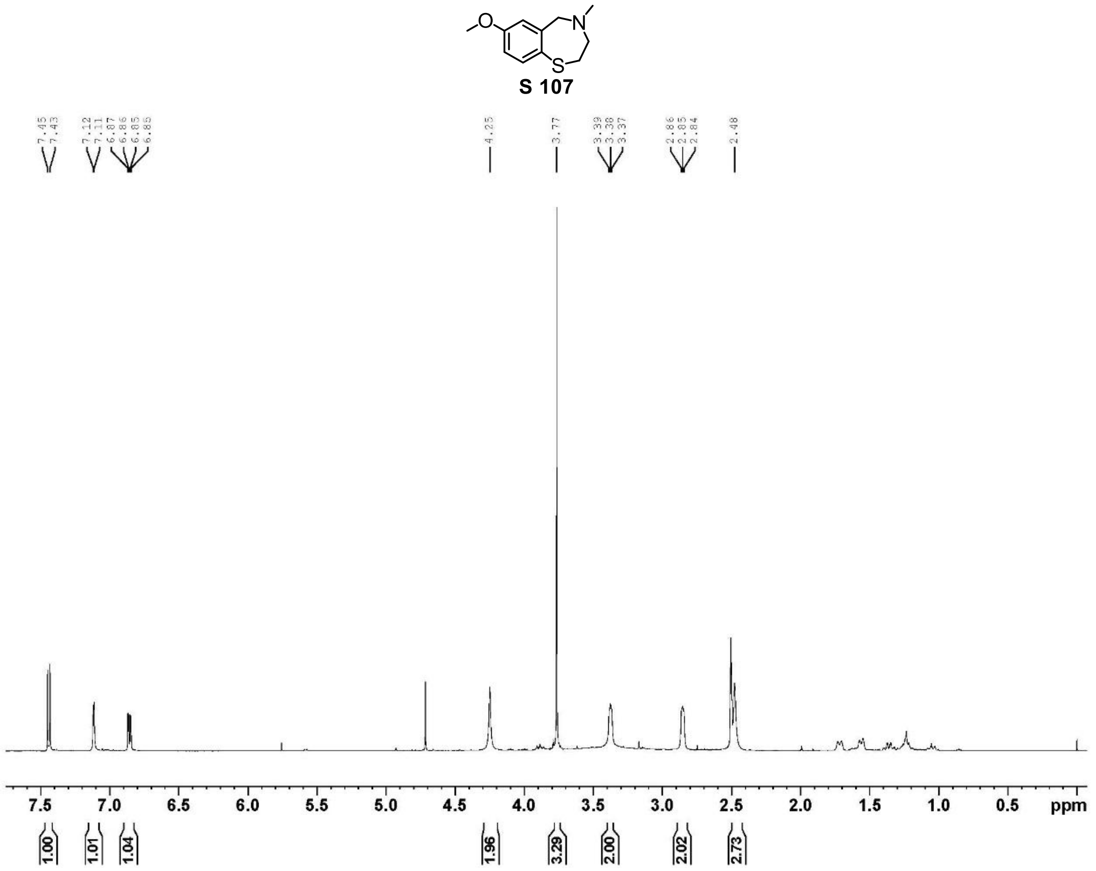

Figure 9: ^{1}H-NMR of **S107**.

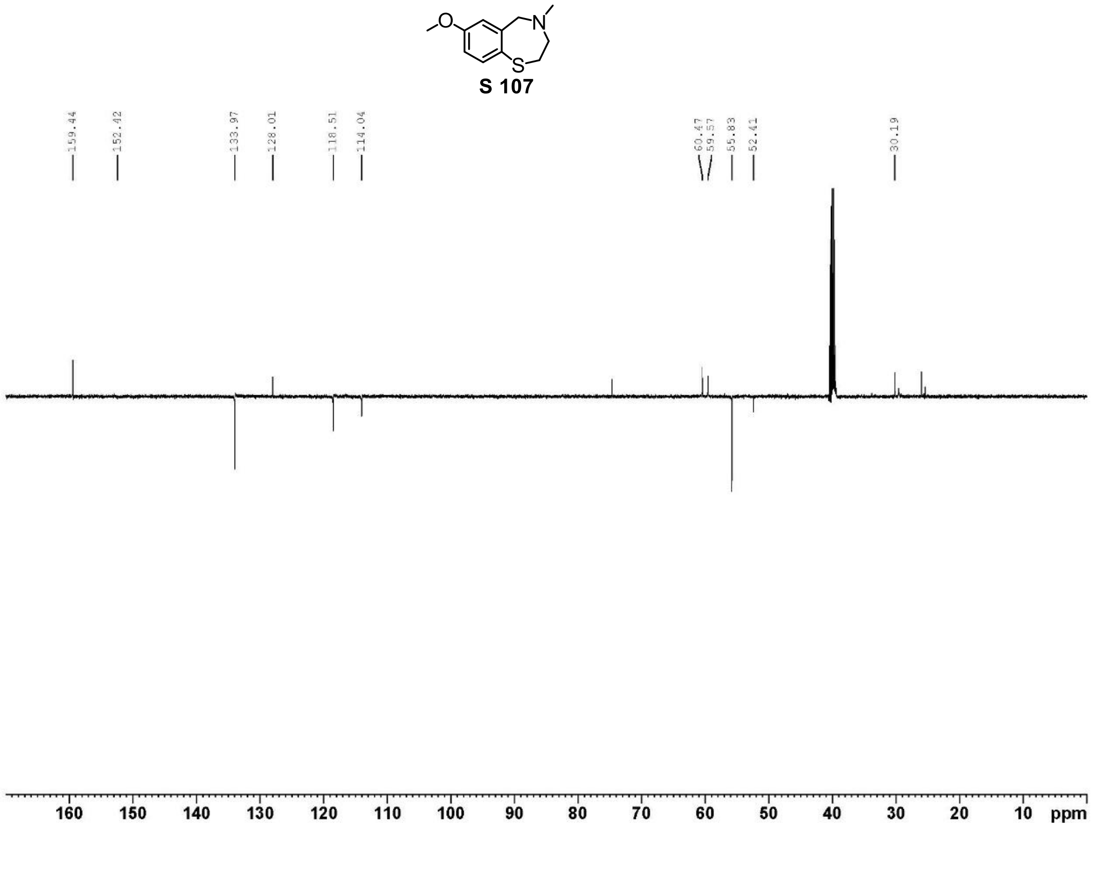

Figure 10: ^{13}C-NMR of **S107**.

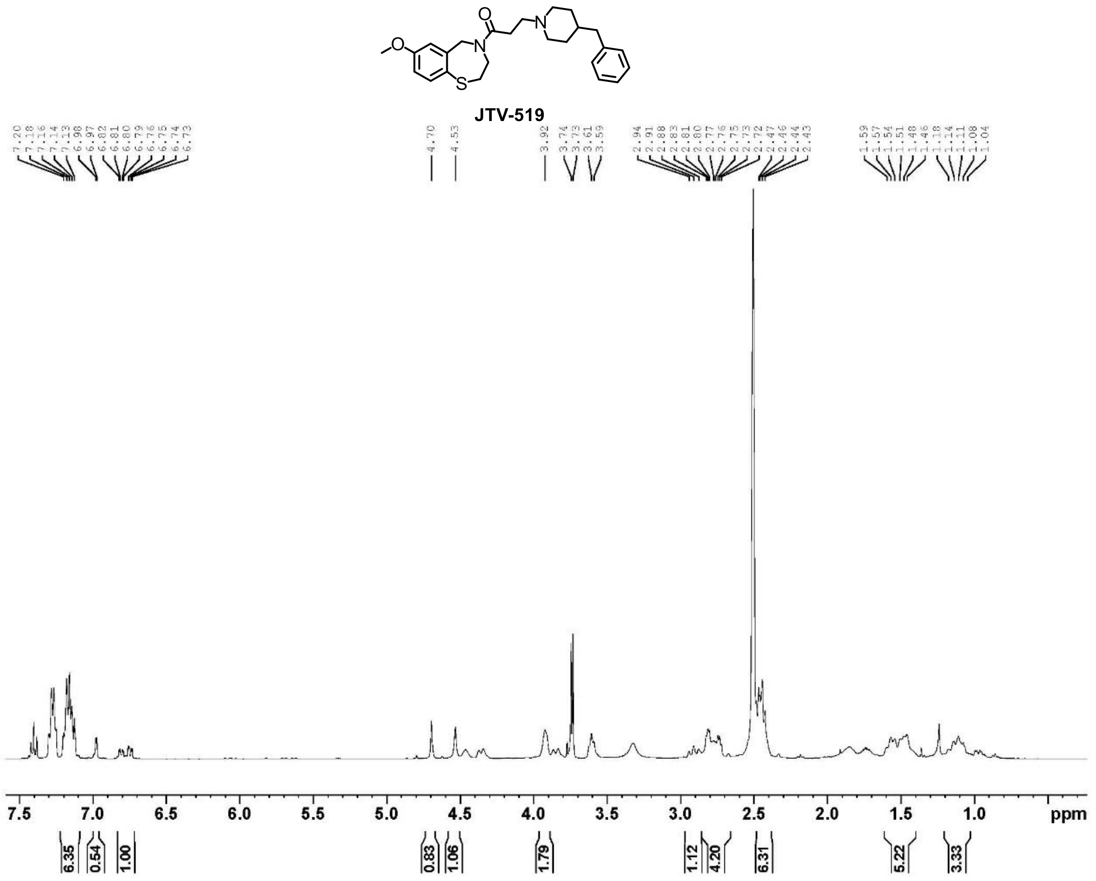

Figure 11^{1}H-NMR of **JTV-519**.

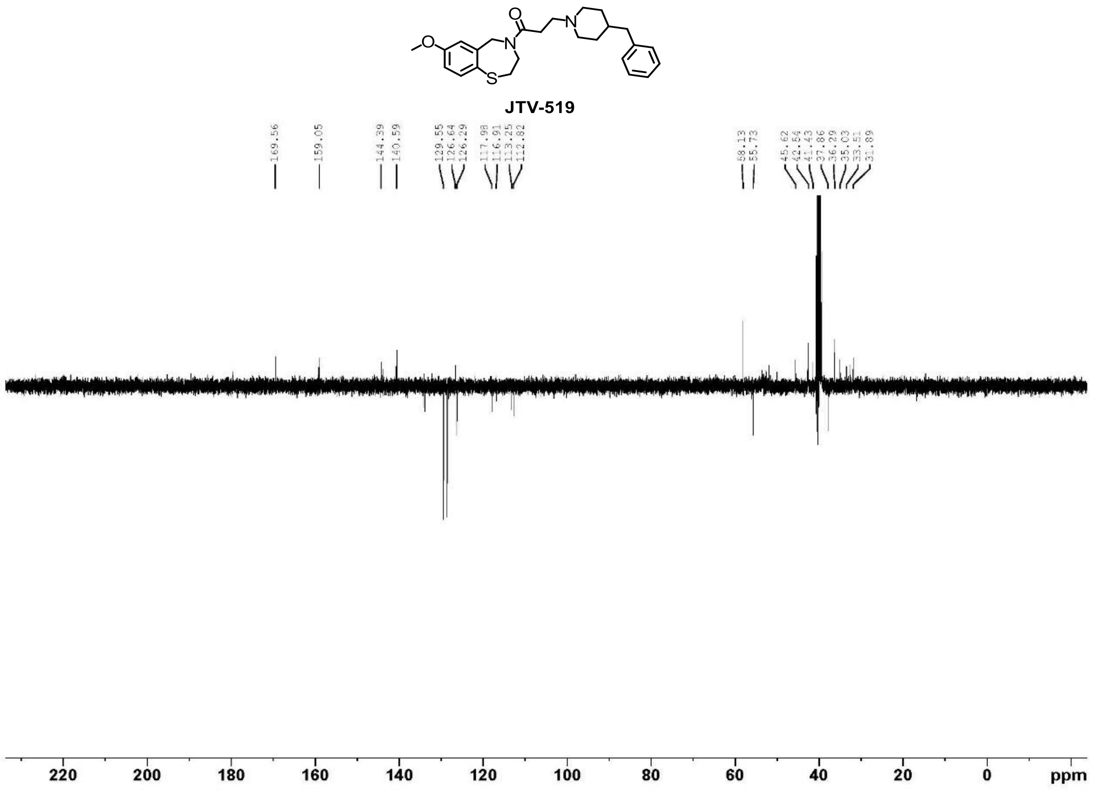

Figure 12: ^{13}C-NMR of **JTV-519**.

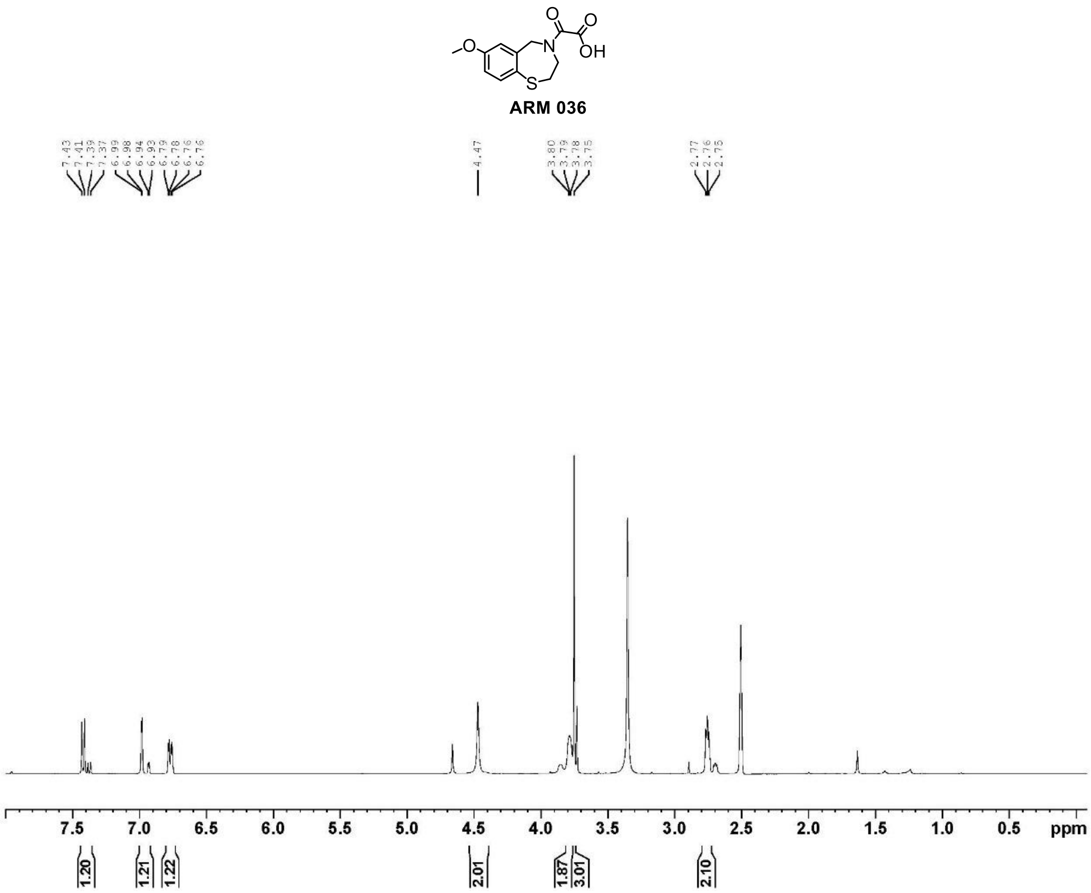

Figure 13: ^{1}H-NMR of **ARM 036**.

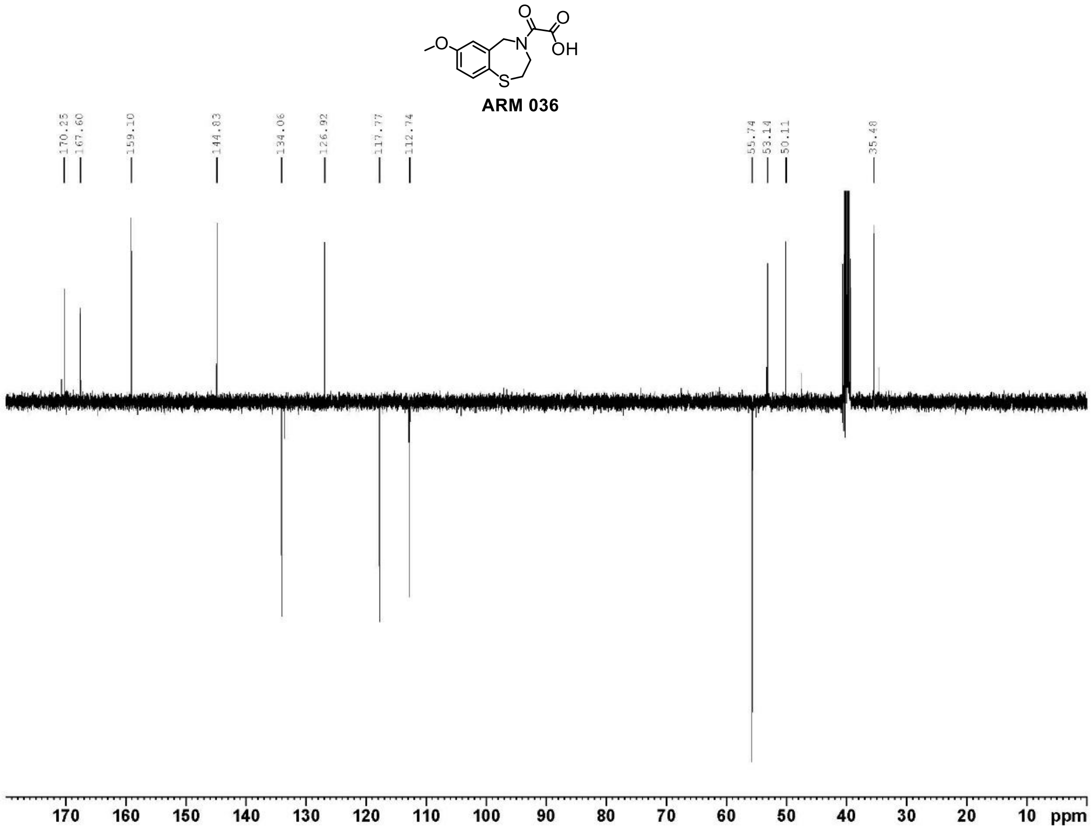

Figure 14: ^{13}C-NMR of **ARM 036**.

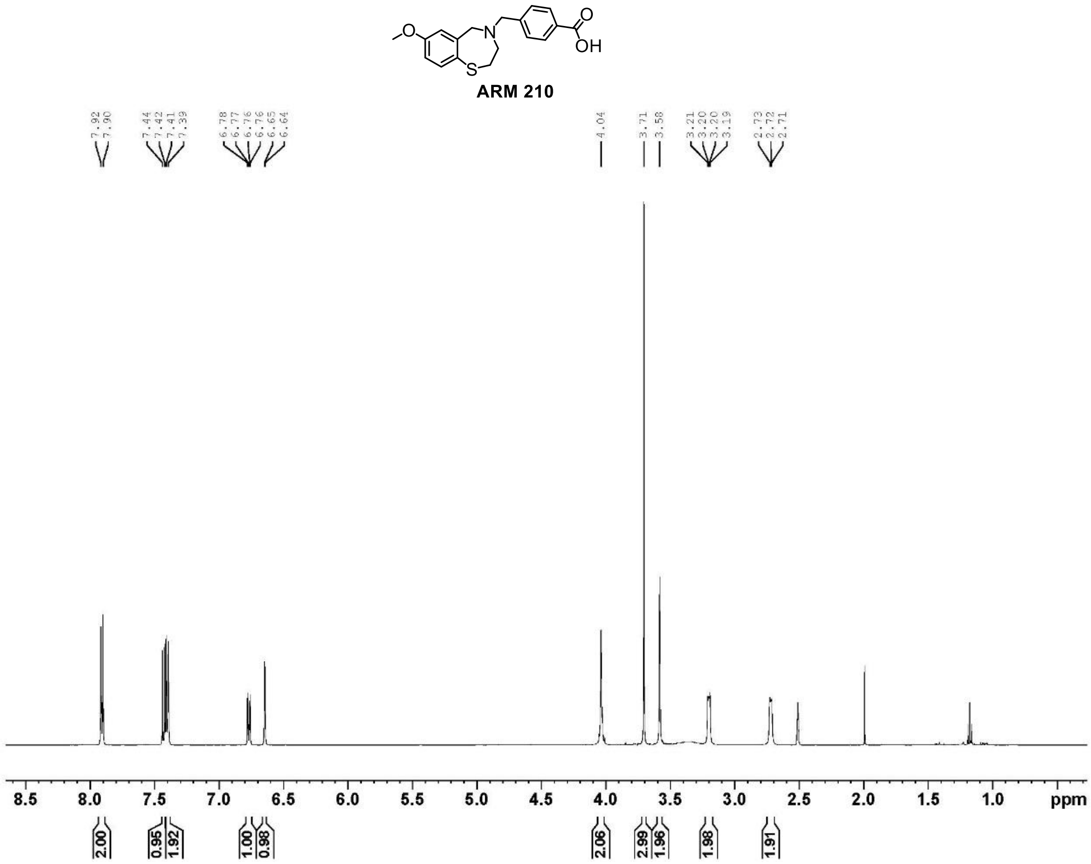

Figure 15: ^{1}H-NMR of **ARM 210**.

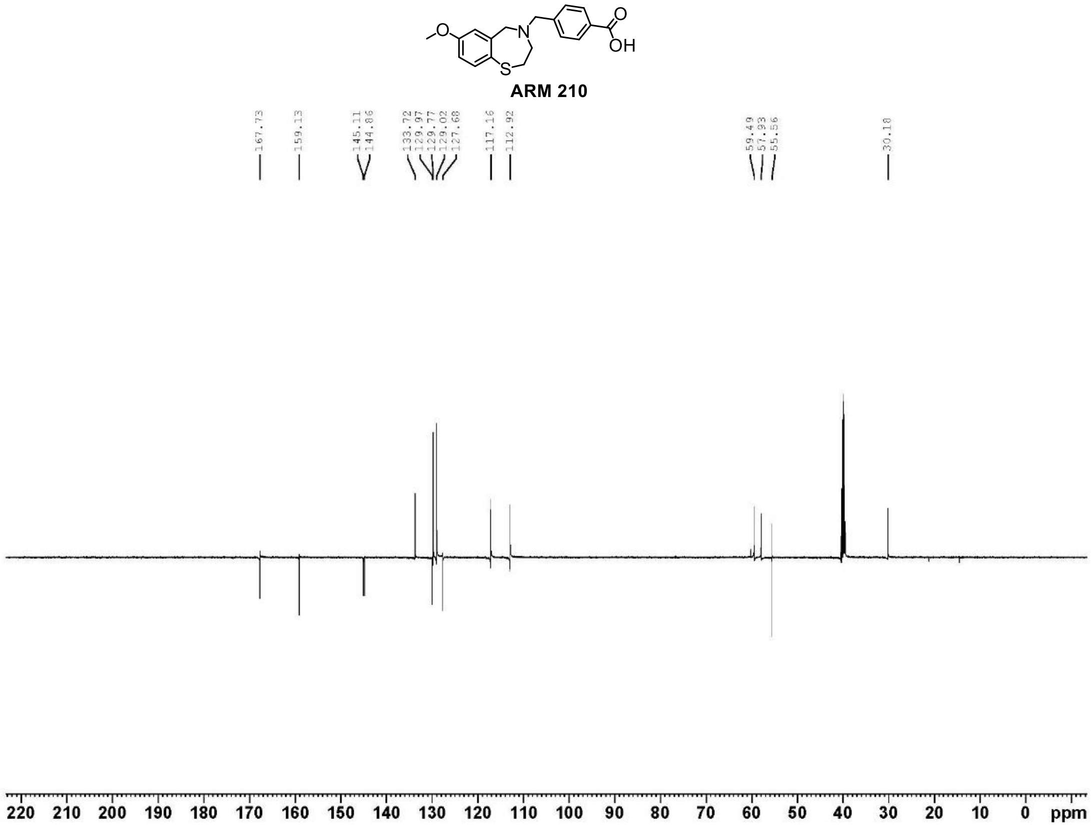

Figure 16: ^{13}C-NMR of **ARM 210**.

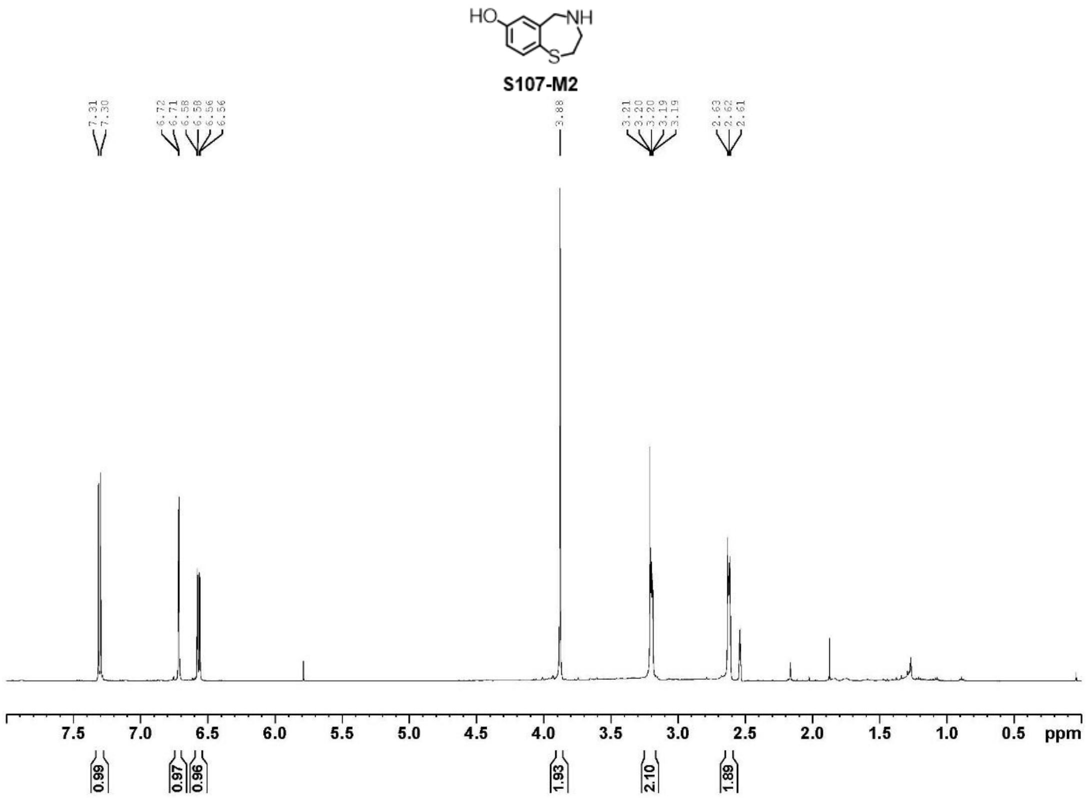

Figure 17: ^{1}H-NMR of **S107-M2**.

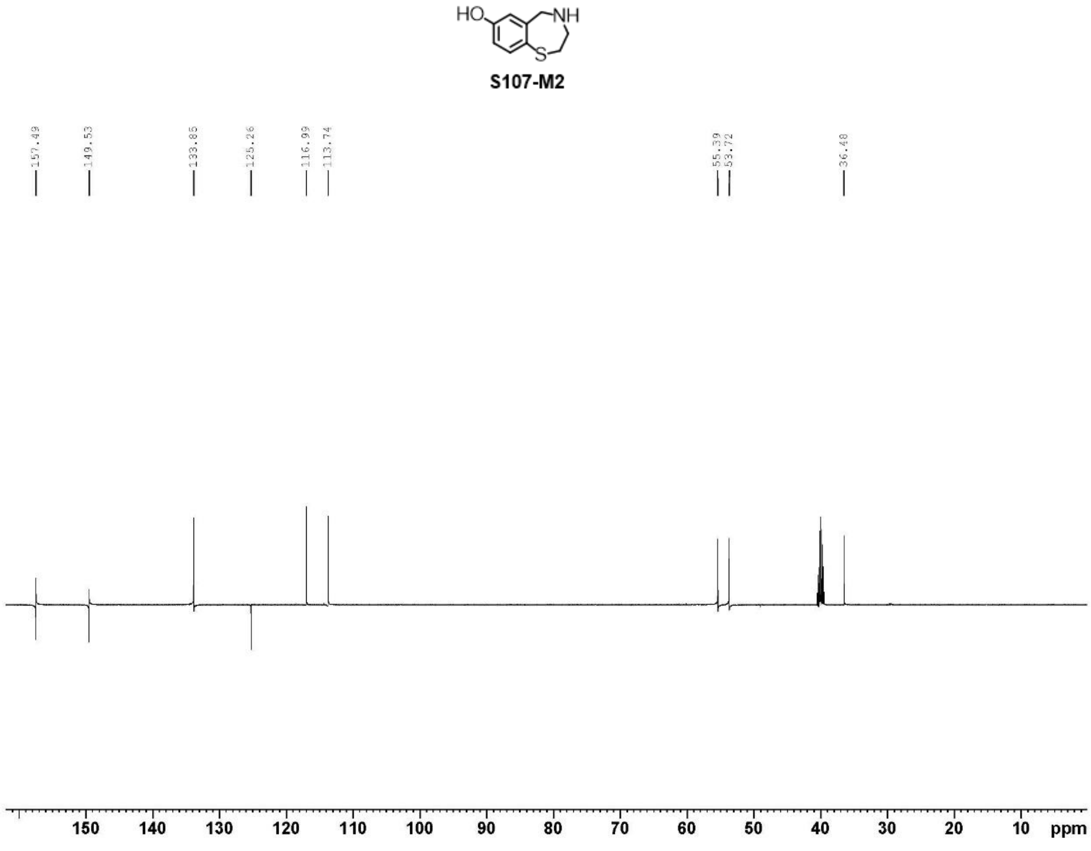

Figure 18: ^{13}C-NMR of **S107-M2**.

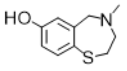

S107-M1b

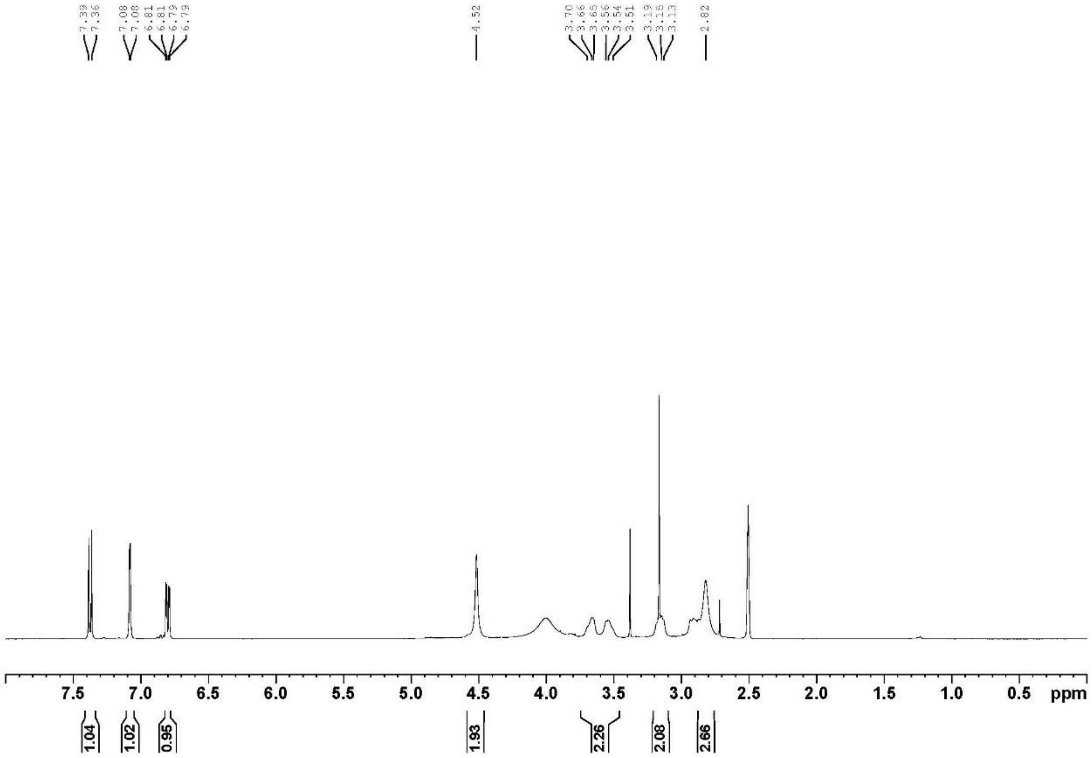

Figure 19^{1}H-NMR of **S107-M1b**.

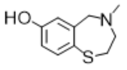

S107-M1b

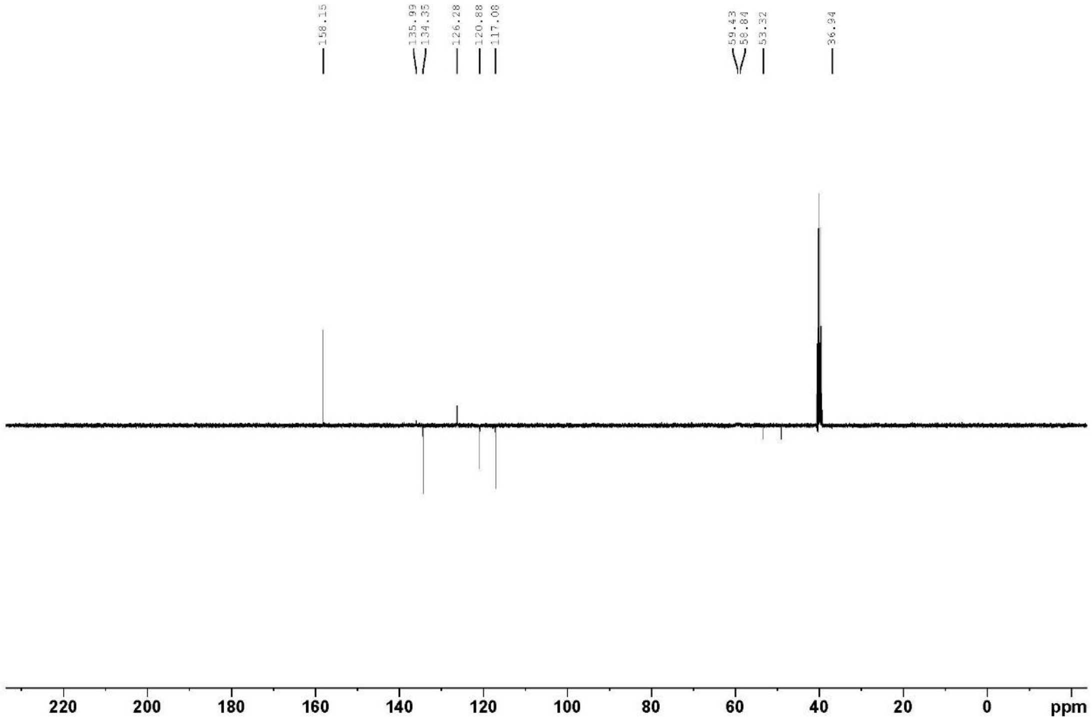

Figure 20: 13C-NMR of **S107-M1b**.

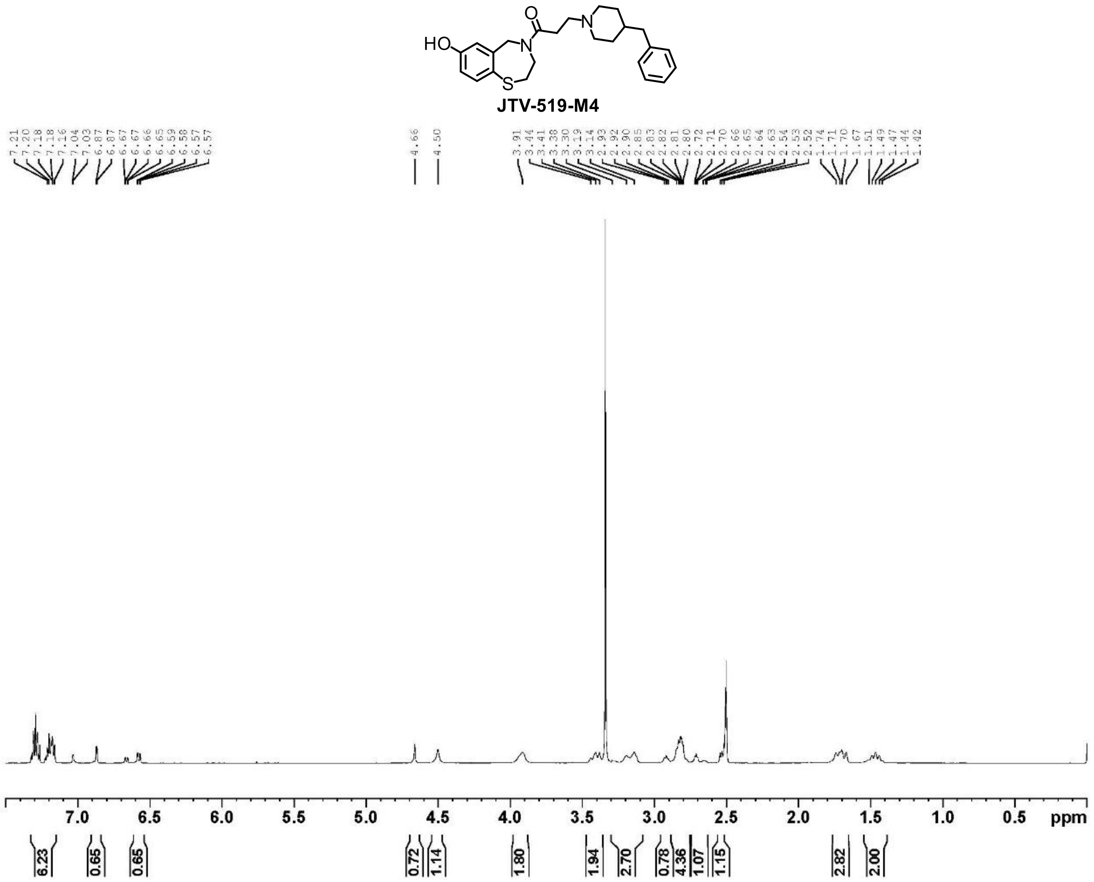

Figure 21: ^{1}H-NMR of **JTV-519-M4**.

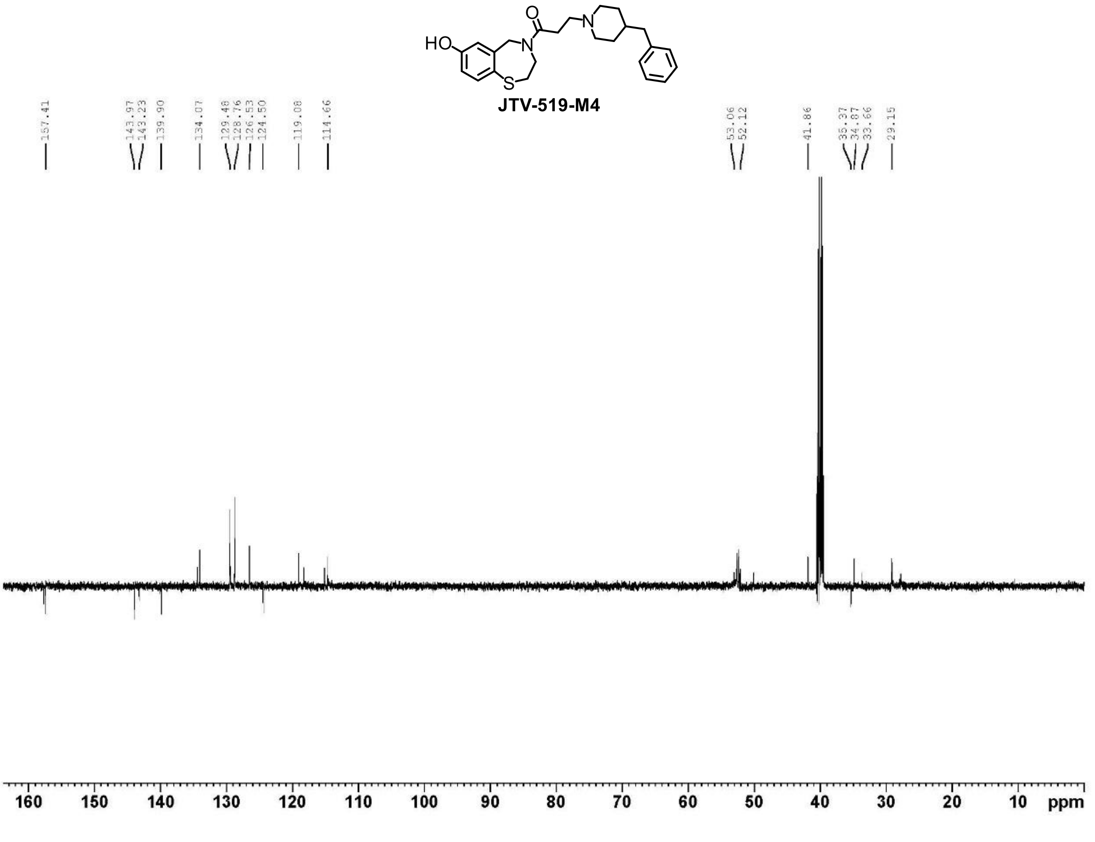

Figure 22 : ^{13}C-NMR of **JTV 519-M4.**

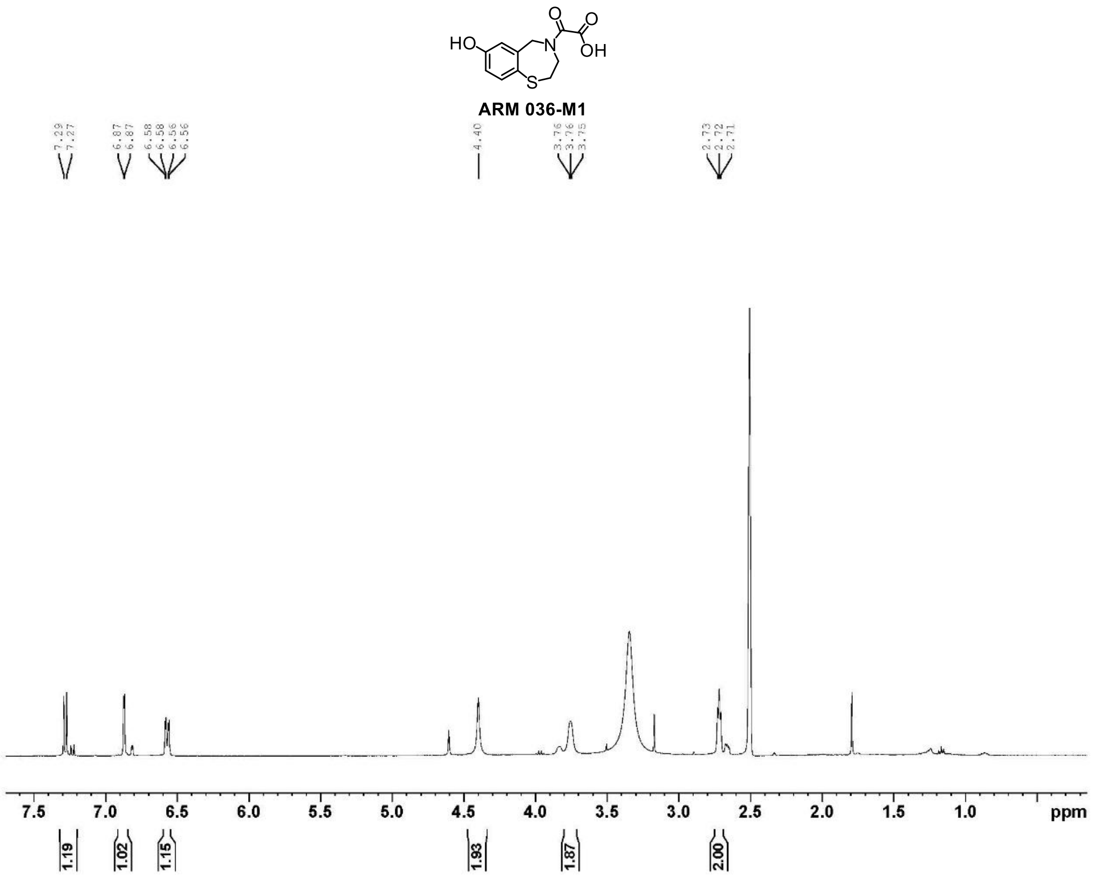

Figure 23: ^{1}H-NMR of **ARM 036-M1**.

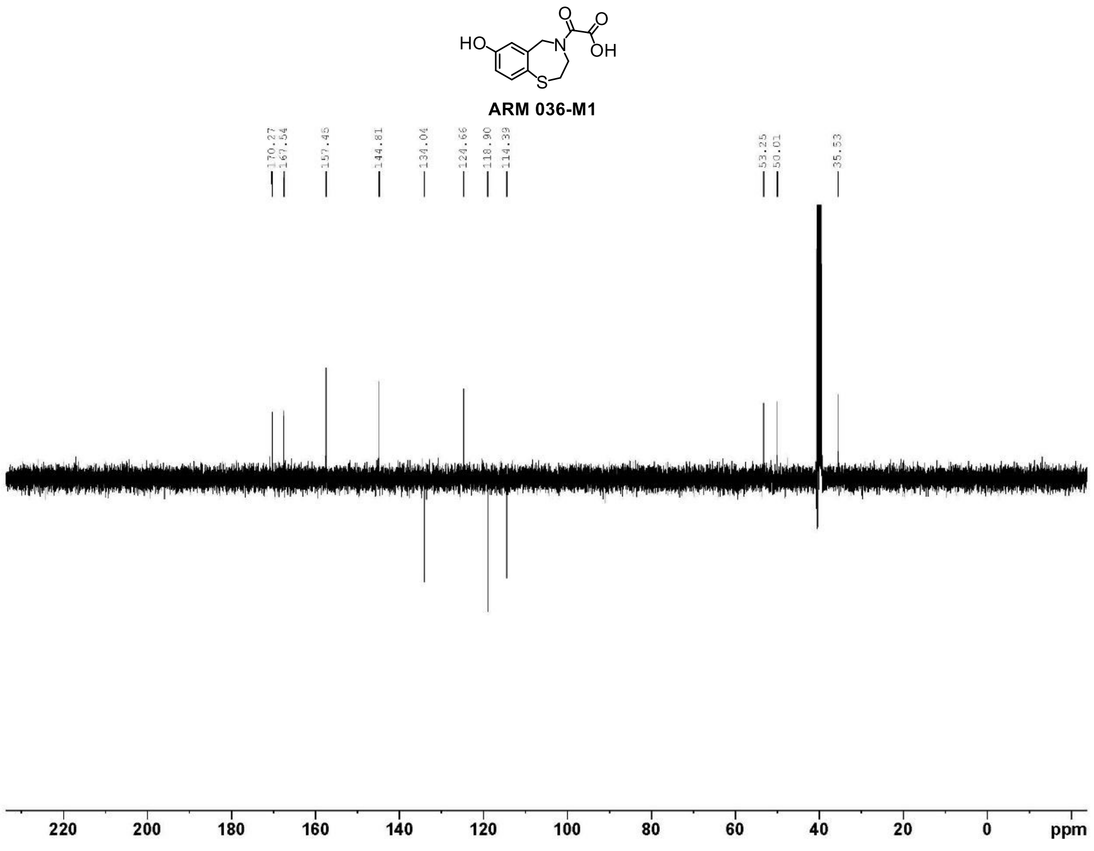

Figure 24: ^{13}C-NMR of **ARM 036-M1**.

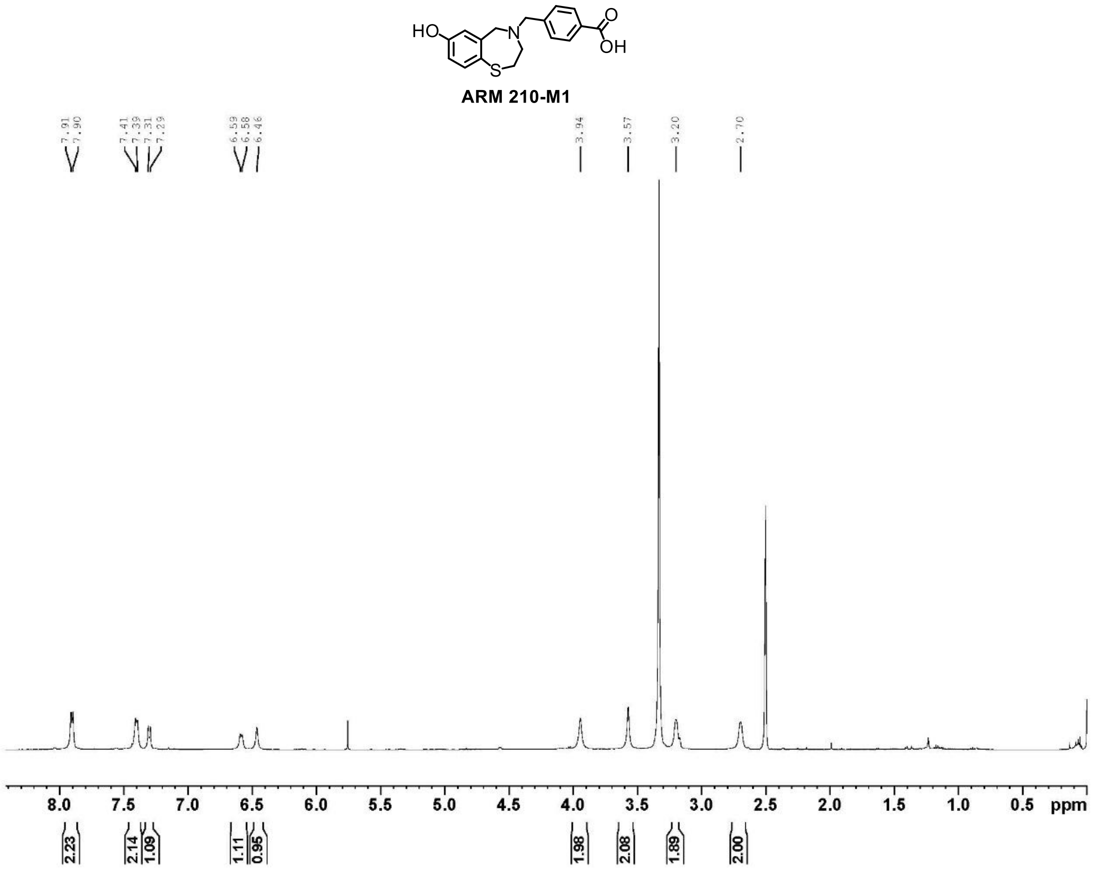

Figure 25: ^{1}H-NMR of **ARM 210-M1**.

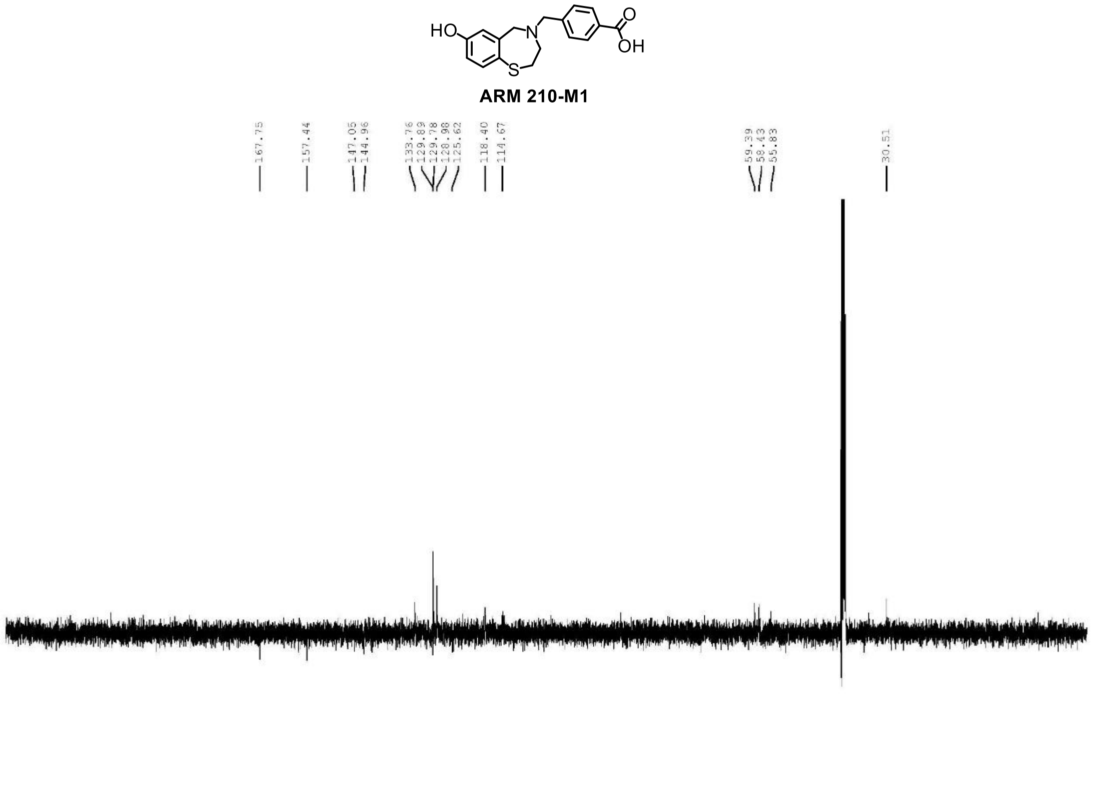

Figure 26: ^{13}C-NMR of **ARM 210-M1**.
